# Supplementary material for: Is there anything good about conspiracy beliefs? Belief in COVID-19 conspiracy theories is associated with benefits to well-being
Source: PLoS One. 2025 Mar 21;20(3):e0319896. doi: 10.1371/journal.pone.0319896 (PMC11927887; doi:10.1371/journal.pone.0319896)
Supplement: S1 Data — (DOCX) [file pone.0319896.s001.docx]

**Supplemental Material**

This Supplemental Material presents a number of analyses that expand upon and buttress portions of the main text. First, we present a factor analysis to determine the extent to which the measure of COVID-19 conspiracy beliefs employed in Study 1 is separable from the measure of conspiracist ideation. Second, we report the results of a factor analysis examining the latent factor structure of the well-being measures in Wave 5. Third, we present a set of analyses in which we remove an item from the Generic Conspiracist Beliefs Scale (GCBS; Brotherton et al., 2013), due to its overlap with the content of our measure of COVID-19 conspiracy beliefs, to ensure the robustness of the results presented in Study 1 of the main text. Fourth, we present a series of analyses in which each item assessing COVID-19 conspiracy beliefs is substituted for the composite measure of COVID-19 conspiracy beliefs as a variable in the regressions analyses predicting stress and well-being. We do this for both Study 1 and Study 2. Fifth, we report the results of analyses that use structural equation modelling (SEM) to replicate the results of our analyses using multiple regression. Lastly, we report the results of supplementary analyses in which we include political ideology, age, race, income, and education as additional covariates in our key multiple regression and propensity score weighting models to ensure that these covariates do not account for the effects reported.

**Factor Analysis of COVID-19 Conspiracy Belief and Conspiracist Ideation Measures**

A central idea in the theoretical framework that drives this research is that belief in *an event conspiracy theory* is related to a person’s general tendency to believe conspiracy theories, but the two variables are nonetheless separable constructs. To put this idea to the test, we decided to run a factor analysis involving our measures of COVID-19 conspiracy beliefs and conspiracist ideation. Before conducting this analysis, however, we assessed the adequacy of our data for the technique. The Kaiser-Meyer-Olkin measure of sampling adequacy was 0.94—above the recommended standard—and Bartlett’s test of sphericity was significant, *χ*2(66) = 3,983.20, *p* < .0001, both of which indicated that our data was indeed suitable for factor analysis.

Next, we entered two items from the measure of COVID-19 conspiracy beliefs and ten items assessing conspiracist ideation from the Generic Conspiracist Beliefs Scale (Brotherton et al., 2013) into a model that used principal axis factoring with a direct oblimin (oblique) rotation, and requested two factors be extracted. The two factors showed a significant positive correlation, *r*(439) = 0.66, *p* < 0.001. As seen in Supplemental Table 1, the items assessing conspiracist ideation loaded strongly on the first factor, which accounted for 59.8% of the variance. The second factor consisted of the two items assessing COVID-19 conspiracy beliefs, and it accounted for an additional 5.92% of the variance.

**Supplemental Table 1**

*Factor Analysis of Measures of COVID-19 Conspiracy Beliefs & Conspiracist Ideation*

| Item | Factor | |
| --- | --- | --- |
|  | Conspiracist ideation | COVID-19 conspiracy beliefs |
| The government permits or perpetrates acts of terrorism on its own soil, disguising its involvement. | .92 |  |
| The government is involved in the murder of innocent citizens and/or well-known public figures, a... | .86 |  |
| New and advanced technology which would harm current industry is being suppressed. | .76 |  |
| A small, secret group of people is responsible for making all major world decisions, such as goin... | .76 |  |
| Certain significant events have been the result of the activity of a small group who secretly man... | .75 |  |
| Experiments involving new drugs or technologies are routinely carried out on the public without t..  . | .74 |  |
| Some UFO sightings and rumors are planned or staged in order to distract the public from real ali... | .60 |  |
| Secret organizations communicate with extraterrestrials, but keep this fact from the public. | .55 |  |
| The spread of certain viruses and/or diseases is the result of the deliberate, concealed efforts... | .55 |  |
| Groups of scientists manipulate, fabricate, or suppress evidence in order to deceive the public. | .48 |  |
| COVID-19 is intentionally presented as dangerous in order to mislead the public. |  | .88 |
| COVID-19 is intentionally presented as dangerous in order to mislead the public. |  | .83 |
| *Note:* Factor loadings below .40 are suppressed. |  |  |

**Factor Structure of Well-Being Measures in Wave 5 of Study 2**

Study 2 used factor analysis to determine the factors underlying the various measures of well-being. The full results of the factor analysis of the Wave 3 data are reported in the main text. The factor structure of the Wave 5 data strongly resembled those of Wave 3. As seen in Supplemental Table 2, the first factor consisted of items dealing with depression and anxiety symptomology, and accounted for 56.4% of the variance. The second factor accounted for an additional 6.5% of the variance, and was comprised of items assessing happiness and hopefulness. In keeping with Wave 3 data, we labelled the first factor negative symptomology and the second factor contentment.

**Supplemental Table 2**

*Results from a Factor Analysis of Measure of Subjective Well-Being in Wave 5*

| Item | Factor | |
| --- | --- | --- |
|  | Negative symptomology | Contentment |
| Trouble relaxing | .93 |  |
| Not being able to stop or control worrying | .92 |  |
| Feeling nervous, anxious or on edge | .91 |  |
| Worrying too much about different things | .89 |  |
| Being so restless that it is hard to sit still | .84 |  |
| Feeling afraid as if something awful might happen | .80 |  |
| Feeling down, depressed, or hopeless | .78 |  |
| Little interest or pleasure in doing things | .76 |  |
| Becoming easily annoyed or irritable | .74 |  |
| Trouble concentrating on things, such as reading the newspaper or watching television | .73 |  |
| Feeling tired or having little energy | .70 |  |
| Feeling bad about yourself - or that you are a failure or have let yourself or your family down | .69 |  |
| Poor appetite or overeating | .67 |  |
| Trouble falling or staying asleep, or sleeping too much | .66 |  |
| Moving or speaking so slowly that other people have noticed? Or the opposite - being so fidgety or restless that you have been moving around more than usual | .61 |  |
| Thoughts that you would be better off dead or of hurting yourself in some way | .59 |  |
| I feel that it is possible to reach the goals I would like to strive for |  | .89 |
| The future seems to me to be hopeful and I believe that things are changing for the better |  | .84 |
| Overall, how happy did you feel yesterday, where 0 is *‘not at all happy’* and 10 is *‘completely happy’*? |  | .53 |

**Removing Item with Content Overlap from Generic Conspiracist Beliefs Scale**

The measure of conspiracist ideation employed in Study 1—the GCBS—contains a series of statements that represent generic versions of the beliefs that undergird a variety of real-world conspiracy beliefs. One item in particular taps into the idea that viruses are deliberately released into the wild for nefarious purposes (“The spread of certain viruses and/or diseases is the result of the deliberate, concealed efforts of some organization”). The overlap in the content between this item and that of the item assessing people’s belief that the COVID-19 pandemic was human-made could pose a problem for the analyses presented in the main text. To rule out this issue, we ran a series of analyses mirroring those in the main text, but instead calculated people’s level of conspiracist ideation when excluding this potentially problematic item.

As before, all variables were standardized and analysed using hierarchical OLS regression. In an initial step, we regressed stress on conspiracist ideation and belief in COVID-19 conspiracy theories. Consistent with the analyses presented in the main text, there was a positive association between conspiracist ideation and stress that was marginally significant, *β* = 0.13 (95% CI: -0.0004, 0.25) *t*(438) = 1.91, *p* = 0.06, and a significant negative relation between belief in COVID-19 conspiracy theories and stress, *β* = -0.15 (95% CI: -0.28, -0.02) *t*(438) = ‑2.25, *p* = 0.03. Similarly, belief in COVID-19 conspiracy theories remained a significant negative predictor of stress when the experience of negative economic consequences due to the pandemic was added to the model, *β* = -0.24 (95% CI: -0.36, -0.11) *t*(437) = -3.63, *p* < 0.001. Clearly, then, the inclusion of the item that shares content with our measures of COVID-19 conspiracy beliefs in the GCBS does not in any way alter the conclusions reported in the main text.

**Using Individual COVID-19 Conspiracy Belief Items as Predictors of Stress and Well-Being**

One potential concern regarding our composite measure of COVID-19 conspiracy beliefs consisting of two items is that the effect is primarily driven by one of the items in the scale. In addition, some people may not regard an item we have included in the composite measure of COVID-19 of conspiracy beliefs as truly tapping into a conspiracy theory relative to other kinds of misinformation. To address these concerns, we present a series of analyses in which each single item assessing COVID-19 conspiracy beliefs is substituted for the composite measure of COVID-19 conspiracy beliefs as a variable in the regressions analyses predicting stress and well-being. Having already generally established the relation between our other covariates—economic turmoil and conspiracist ideation—and well-being, we focus on the relation between each individual COVID-19 conspiracy belief item and well-being in the analyses reported below.

***Study 1***

All variables were standardized prior to analysis. We used a hierarchical Ordinary Least Squares (OLS) regression models to test our hypothesis regarding the relation between individual items of the COVID-19 conspiracy beliefs scale and stress. To start, we regressed people’s reports of general stress on their endorsement of the idea that the COVID-19 pandemic was a hoax (“COVID-19 is intentionally presented as dangerous in order to mislead the public”). In a subsequent step, we added reports of economic turmoil due to the pandemic to the model. Next, we replaced economic turmoil with conspiracist ideation. Lastly, we entered both economic turmoil and conspiracist ideation into the model along with people’s belief that COVID-19 was a hoax. The initial step revealed no significant relation between stress and the belief that COVID-19 was a hoax. However, when economic turmoil and conspiracist ideation were entered into the model, either individually or together, the belief that COVID-19 was a hoax now significantly predicted lower levels of general stress (see Supplemental Table 3).

Next, we repeated the same procedure, but substituted in the belief that COVID-19 was human-made (“COVID-19 was intentionally brought into the world for dark purposes”) as the key predictor. Mirroring the results above, the belief that COVID-19 was human-made did not predict people’s level of general stress when entered alone. Importantly, however, when conspiracist ideation and reports of economic consequences due to the pandemic were entered into the model, either alone or together, the belief that COVID-19 was a human-made now significantly predicted lower levels of general stress (see Supplemental Table 3).

**Supplemental Table 3**

*Regression statistics predicting stress from each of the two COVID-19 conspiracy theory items*

| Predictor | | | *β* | 95% CI | *t* | *p* |
| --- | --- | --- | --- | --- | --- | --- |
| Hoax Item | Simple Relation | | -0.06 | [-0.16, 0.03] | -1.31 | 0.19 |
|  | Controlling for | Economic Turmoil | -0.20 | [-0.30, -0.10] | -3.99 | < 0.001 |
|  |  | Conspiracist Ideation | -0.13 | [-0.25, -0.01] | -2.06 | 0.04 |
|  |  | Both | -0.22 | [-0.34, -0.10] | -3.50 | < 0.001 |
| Human-made Item | Simple Relation | | -0.06 | [-0.15, 0.04] | -1.23 | 0.22 |
|  | Controlling for | Economic Turmoil | -0.18 | [-0.28, -0.08] | -3.54 | < 0.001 |
|  |  | Conspiracist Ideation | -0.13 | [-0.26, 0.002] | -1.93 | 0.05 |
|  |  | Both | -0.19 | [-0.31, -0.06] | -2.89 | 0.004 |

***Study 2***

**Wave 3.** Once again, we standardized all variables before estimating hierarchical OLS regressions. As seen in Supplemental Table 4, there was no simple relation between the belief that COVID-19 is a hoax (“Coronavirus is actually no more dangerous than the common flu.”) and negative symptomology at Wave 3. Moreover, the relation between the belief that COVID-19 is a hoax and negative symptomology at Wave 3 remained non-significant (and descriptively became weaker) after the addition of economic turmoil by itself, conspiracist ideation by itself, or both variables into the model.

Turning to using contentment as an outcome, our analyses revealed that the belief that COVID-19 was a hoax did not significantly predict contentment either when entered alone or when entered alongside either economic turmoil or conspiracist ideation individually. However, when all three predictors were entered into the model simultaneously, greater belief that COVID-19 was a hoax predicted greater contentment (see Supplemental Table4).

**Supplemental Table 4**

*Regression statistics predicting each DV at Wave 3 from belief that COVID-19 was a hoax*

| DV | Predictor(s) | | *β* | 95% CI | *t* | *p* |
| --- | --- | --- | --- | --- | --- | --- |
| Negative symptomology | Simple Relation | | 0.06 | [-0.01, 0.14] | 1.69 | 0.09 |
|  | Controlling for | Economic Turmoil | 0.04 | [-0.04, 0.11] | 0.99 | 0.32 |
|  |  | Conspiracist Ideation | 0.05 | [-0.03, 0.12] | 1.26 | 0.21 |
|  |  | Both | 0.03 | [-0.05, 0.10] | 0.68 | 0.50 |
| Contentment | Simple Relation | | 0.06 | [-0.02, 0.13] | 1.54 | 0.12 |
|  | Controlling for | Economic Turmoil | 0.07 | [-0.001, 0.15] | 1.92 | 0.06 |
|  |  | Conspiracist Ideation | 0.06 | [-0.01, 0.14] | 1.65 | 0.10 |
|  |  | Both | 0.08 | [0.0001, 0.15] | 1.96 | 0.049 |

Turning to the other item in our COVID-19 conspiracy belief scale, the results revealed that the belief that COVID-19 was human-made did not significantly predict negative symptomology in any model estimated (see Supplemental Table 5). Descriptively, the size of the regression coefficient for the relation between the belief that COVID-19 was human-made and negative symptomology shrank in size as covariates were added to the model.

By contrast, the belief that COVID-19 was human-made significantly predicted contentment, both when entered into the model alone and when covariates were added to the model. Descriptively, the regression coefficient became stronger when covariates were added to the model (see Supplemental Table 5).

**Supplemental Table 5**

*Regression statistics predicting each DV at Wave 3 from belief that COVID-19 was human-made*

| DV | Predictor(s) | | *β* | 95% CI | *t* | *p* |
| --- | --- | --- | --- | --- | --- | --- |
| Negative symptomology | Simple Relation | | 0.05 | [-0.02, 0.13] | 1.38 | 0.17 |
|  | Controlling for | Economic Turmoil | 0.01 | [-0.06, 0.09] | 0.34 | 0.74 |
|  |  | Conspiracist Ideation | 0.02 | [-0.06, 0.10] | 0.54 | 0.59 |
|  |  | Both | -0.01 | [-0.09, 0.06] | -0.31 | 0.76 |
| Contentment | Simple Relation | | 0.10 | [0.03, 0.17] | 2.69 | 0.007 |
|  | Controlling for | Economic Turmoil | 0.12 | [0.05, 0.20] | 3.25 | 0.001 |
|  |  | Conspiracist Ideation | 0.11 | [0.04, 0.19] | 2.96 | 0.003 |
|  |  | Both | 0.13 | [0.06, 0.21] | 3.42 | < 0.001 |

**Wave 5.** We then repeated the same analyses for the negative symptomology and contentment variables measured at Wave 5, starting with the belief that COVID-19 was a hoax. Standing in contrast to Wave 3, the belief that COVID-19 was a hoax significantly predicted negative symptomology when entered alone as a predictor in the model. Belief that COVID-19 was a hoax remained a significant positive predictor of negative symptomology when conspiracist ideation was entered into the model, but this conspiracy belief no longer predicted negative symptomology was no longer a significant predictor when economic turmoil was entered into the models (see Supplemental Table 6).

**Supplemental Table 6**

*Regression statistics predicting each DV at Wave 5 from belief that COVID-19 was a hoax*

| DV | Predictor(s) | | *β* | 95% CI | *t* | *p* |
| --- | --- | --- | --- | --- | --- | --- |
| Negative symptomology | Simple Relation | | 0.09 | [0.02, 0.17] | 2.45 | 0.01 |
|  | Controlling for | Economic Turmoil | 0.06 | [-0.008, 0.14] | 1.74 | 0.08 |
|  |  | Conspiracist Ideation | 0.08 | [0.002, 0.15] | 2.03 | 0.04 |
|  |  | Both | 0.05 | [-0.02, 0.12] | 1.44 | 0.15 |
| Contentment | Simple Relation | | -0.002 | [-0.08, 0.07] | -0.06 | 0.95 |
|  | Controlling for | Economic Turmoil | 0.02 | [-0.06, 0.09] | 0.48 | 0.64 |
|  |  | Conspiracist Ideation | 0.004 | [-0.07, 0.08] | 0.10 | 0.92 |
|  |  | Both | 0.02 | [-0.05, 0.10] | 0.56 | 0.58 |

Lastly, we used the belief that COVID-19 was human-made as a focal predictor (see Supplemental Table 7). Across all models estimated, the belief that COVID-19 was human-made was not a significant predictor of either negative symptomology or contentment.

**Supplemental Table 7**

*Regression statistics predicting each DV at Wave 5 from belief that COVID-19 was human-made*

| DV | Predictor(s) | | *β* | 95% CI | *t* | *p* |
| --- | --- | --- | --- | --- | --- | --- |
| Negative symptomology | Simple Relation | | 0.05 | [-0.02, 0.13] | 1.38 | 0.17 |
|  | Controlling for | Economic Turmoil | 0.01 | [-0.06, 0.09] | 0.34 | 0.74 |
|  |  | Conspiracist Ideation | 0.02 | [-0.06, 0.10] | 0.54 | 0.59 |
|  |  | Both | -0.01 | [-0.09, 0.06] | -0.31 | 0.76 |
| Contentment | Simple Relation | | 0.01 | [-0.07, 0.08] | 0.18 | 0.86 |
|  | Controlling for | Economic Turmoil | 0.03 | [-0.04, 0.11] | 0.92 | 0.36 |
|  |  | Conspiracist Ideation | 0.02 | [-0.06, 0.10] | 0.50 | 0.62 |
|  |  | Both | 0.04 | [-0.03, 0.12] | 1.11 | 0.27 |

This set of analyses provides evidence that the results presented in the main text generalize to the two different specific conspiracy beliefs about COVID-19. That is, both items contribute to the effects reported in the main text. Beyond speaking to the robustness of our results, these findings also underscore that a variety of conspiracy beliefs can provide benefits to believers.

**Structural Equation Models**

In light of concerns about inflated Type I error rates when using multiple regression (Westfall & Yarkoni, 2016) and the use of factor scores as outcomes in Study 2, we replicated our multiple regression analyses using SEM through the *lavaan* package in R (v. 4.2.2). In Study 1, we predicted stress from belief in COVID-19 conspiracy theories (a latent variable consisting of the two COVID-19 conspiracy belief items), the experience of turmoil, and conspiracist ideation (a latent variable consisting of ten items from the Generic Conspiracist Beliefs Scale). The model was estimated using Maximum Likelihood (ML) with the NLMINB optimization method and included 29 parameters, based on data from 495 observations. The chi-square test for the user model was significant, χ²(75) = 538.859, p < .001, indicating that the model fit could be improved. The Comparative Fit Index (CFI) was 0.906, suggesting an acceptable fit. However, the Tucker-Lewis Index (TLI) was slightly below the commonly accepted threshold, at 0.886, indicating some concerns about model fit. The results revealed that while conspiracist ideation showed no relation to stress, *β =* 0.01, *z* = 1.17, *p* = 0.24, experiencing economic turmoil was associated with greater stress, *β =* 0.14, *z* = 7.12, *p* < 0.001. Importantly, we found that belief in COVID-19 conspiracy theories was significantly associated with less stress, *β =* -0.14, *z* = 3.25, *p* = 0.001.

For Study 2, we first sought to test whether a model in which well-being at Wave 3 was specified as being a single latent factor under which all constituent outcome measures (i.e., depression, anxiety, happiness) were nested had better fit than a model in which we specified two latent variables—one for negative symptomology and one for contentment. The model involving two latent factors demonstrated a lower AIC (58,195.468) and BIC (58,506.097) compared to the model in which only a single latent well-being factor was specified, which yielded an AIC of 60,021.216 and a BIC of 60,313.573. This finding lends support to our decision to model our outcomes using factor scores representing negative symptomology and contentment.

Regarding the substantive results, we focused on analyzing contentment as an outcome, both at Wave 3 and 5. At Wave 3, the experience of economic turmoil was associated with less contentment, *β =* -0.03, *z* = -3.24, *p* = 0.001, while bore no relation to contentment, *β =* -0.04, *z* = -1.29, *p* = 0.20. Notably, greater belief in COVID-19 conspiracy theories was associated with greater contentment, *β =* 0.07, *z* = 2.59, *p* = 0.01. At Wave 5, contentment was not significantly predicted by either belief in COVID-19 conspiracy theories, *β =* 0.02, *z* = 0.83, *p* = 0.41, or conspiracist ideation, *β =* -0.02, *z* = -0.66, *p* = 0.51. The experience of economic turmoil continued to predict contentment at Wave 5, *β =* -0.06, *z* = -4.94, *p* < 0.001.

To provide an additional test of whether there was a significant decline in the strength of the relation between COVID-19 conspiracy beliefs and contentment over time, we used a chi-squared test to compare the fit of a model in which the coefficients for belief in COVID-19 conspiracy theories at Waves 3 and 5 were allowed to vary freely versus one in which they were set to be equivalent. The results revealed that these models were significantly different, Δχ²(1) = 4.19, *p* = 0.04, suggesting that the strength of the relation between COVID-19 conspiracy beliefs and contentment declines over time.

**Introducing Political Ideology and Demographic Variables as Robustness Checks**

The theoretical framework guiding our research posits that the experience of turmoil and conspiracist ideation must be taken into account when attempting to isolate the effect of believing event conspiracy theories on well-being. We focused on these variables because they tend to have robust associations with belief in event conspiracy theories and well-being (e.g., Bayliss et al., 2017; Douglas et al., 2017; 2019; Farhart et al., 2021; Freeman & Bentall, 2017; Swami et al., 2017; see “Current Research” in the main text for full details). The results of our analyses consistently support our hypotheses.

However, readers may wonder whether variables that are not included in our model may also account for these relations. We elected not to include other variables that may on the surface seem relevant, like political ideology and demographic characteristics, because they either do not show consistent relations with both our key focal predictor and outcome (as in the case of ideology) or are likely proxies for other psychological variables like beliefs, attitudes, or personality characteristics (as in the case of demographics). That said, to ensure that the results we report are robust to the inclusion of additional covariates, we added political ideology, age, race, income, and education to the multiple regression and propensity score weighting analyses reported in the main text.

***Study 1***

**Multiple Regression Analysis.** Using the same analytic strategy employed in the main text, we regressed stress on belief in COVID-19 conspiracy theories, reports of economic turmoil, conspiracist ideation, political ideology (higher values indicate greater conservatism), age (in years), race (0 = White, 1 = Not White), income (annual family income), and education (higher values indicate higher levels of education achieved). As seen in Supplemental Table 8, the experience of economic turmoil significantly predict stress—as before. With regard to the new covariates, only higher educational achievement predicted reduced stress. Importantly, greater belief in COVID-19 conspiracy theories continues to predict less stress, providing yet another demonstration of the robustness of this finding.

**Supplemental Table 8**

*OLS regression predicting stress from expanded list of covariates in Study 1*

| Predictor | *β* | 95% CI | *t* | *p* |
| --- | --- | --- | --- | --- |
| Belief in COVID-19 conspiracy theories | -0.20 | [-0.34, -0.06] | -2.81 | 0.005 |
| Economic turmoil | 0.31 | [0.21, 0.41] | 5.89 | <0.001 |
| Conspiracist ideation | 0.05 | [-0.08, 0.19] | 0.78 | 0.44 |
| Political orientation (higher values = more conservative) | -0.08 | [-0.18, 0.02] | -1.52 | 0.13 |
| Age | 0.02 | [-0.08, 0.11] | 0.31 | 0.76 |
| Race (0 = White, 1 = Not White) | 0.01 | [-0.21, 0.22] | 0.05 | 0.96 |
| Income | -0.04 | [-0.13, 0.06] | -0.72 | 0.47 |
| Education | 0.11 | [0.02, 0.21] | 2.29 | 0.02 |

**Propensity Weighting Analysis.** We repeated the same general procedure of including additional covariates in the form of political ideology and demographic characteristics for the analyses involving propensity score weighting. Specifically, entropy balancing weights were generated via the *WeightIt* and *cobalt* packages in R (v. 4.2.2), which was successful in achieving balance: the correlations between the continuous treatment and covariates all approximated 0. The final sample for this analysis was 162. In our key weighted regression analysis, belief in COVID-19 conspiracy theories significantly predicted stress such that those who endorsed conspiracy theories to a greater extent were less stressed, *β* = -0.11, *SE* = 0.05, *t*(430) = -2.19, *p* = 0.03.

***Study 2***

**Multiple Regression Analysis.** We repeated the procedure above with the regression model demonstrating a significant relation between belief in COVID-19 conspiracy theories and contentment at Wave 3 to ensure that the additional covariates did not render this relation non-significant. Using Wave 3 data, we regressed contentment scores on belief in COVID-19 conspiracy theories, conspiracist ideation, reports of economic turmoil, political ideology (higher values indicate greater conservatism), age (in years), race (0 = White, 1 = Not White), income (weekly family income), and education (0 = did not attend post-secondary school, 1 = attended post-secondary school). The results of this model revealed that greater economic turmoil continued to predict less contentment. Moreover, greater conservatism and higher family income also predicted greater contentment. Of greatest relevance, the results revealed that stronger endorsement of COVID-19 conspiracy theories continued to predict greater contentment at Wave 3—providing converging support for the analyses reported in the main text.

For brevity, we do not report the results of the three other multiple regression analyses presented in the main text—those involving negative symptomology at Wave 3 and 5, as well as contentment at Wave 5—as none of the substantive conclusions are changed by the inclusion of additional covariates (see <https://osf.io/7b3xp/?view_only=f649bb67327c40629d9b63b3b2188fd5> for output corresponding to all outcomes reported in the main text).

**Supplemental Table 9**

*OLS regression predicting contentment at Wave 4 from expanded list of covariates in Study 2*

| Predictor | *β* | 95% CI | *t* | *p* |
| --- | --- | --- | --- | --- |
| Belief in COVID-19 conspiracy theories | 0.14 | [0.07, 0.22] | 3.66 | <0.001 |
| Economic turmoil | -0.10 | [-0.18, -0.03] | -2.65 | 0.01 |
| Conspiracist ideation | -0.01 | [-0.08, 0.06] | -0.27 | 0.79 |
| Political orientation (higher values = more conservative) | 0.09 | [0.01, 0.16] | 2.28 | 0.02 |
| Age | 0.07 | [-0.01, 0.15] | 1.84 | 0.07 |
| Race (0 = White, 1 = Not White) | -0.04 | [-0.30, 0.22] | -0.31 | 0.76 |
| Income | 0.19 | [0.11, 0.26] | 4.96 | <0.001 |
| Education | -0.01 | [-0.16, 0.15] | -0.08 | 0.94 |

**Propensity Score Weighting Analysis.** Lastly, we conducted the propensity score weighting analysis using the analytic strategy described above. Specifically, we used the propensity score weighting technique with contentment at Wave 3 as the outcome, belief in COVID-19 conspiracy theories as a continuous treatment variable, and the experience of economic turmoil, conspiracist ideation, political ideology, age, race, income, and education as confounders. This procedure achieved balance: all correlations between the continuous treatment and covariates approximated 0. The final sample for this analysis was 595 participants. Once again, the weighted linear regression revealed that belief in COVID-19 conspiracy theories significantly predicted greater contentment at Wave 3, *β* = 0.13, *SE* = 0.04, *t*(710) = 3.47, *p* < 0.001.

**References**

Bayliss, D., Olsen, W., & Walthery, P. (2017). Well-being during recession in the UK. *Applied Research in Quality of Life*, *12*, 369-387.

Brotherton, R., French, C. C., & Pickering, A. D. (2013). Measuring belief in conspiracy theories: The generic conspiracist beliefs scale. *Frontiers in Psychology*, *4*, 279.

Douglas, K. M., Sutton, R. M., & Cichocka, A. (2017). The psychology of conspiracy theories. *Current Directions in Psychological Science*, *26*(6), 538-542.

Douglas, K. M., Uscinski, J. E., Sutton, R. M., Cichocka, A., Nefes, T., Ang, C. S., & Deravi, F. (2019). Understanding conspiracy theories. *Political Psychology*, *40*, 3-35.

Farhart, C.E., Miller, J.M., and Saunders, K.L. Conspiracy Stress or Relief? Learned Helplessness and Conspiratorial Thinking. In D.C. Barker & E. Suhay (Eds.). *The Politics of Truth in Polarized America.* Oxford University Press.

Freeman, D., & Bentall, R. P. (2017). The concomitants of conspiracy concerns. *Social Psychiatry and Psychiatric Epidemiology*, *52*(5), 595–604.

Swami, V., Barron, D., Weis, L., Voracek, M., Stieger, S., & Furnham, A. (2017). An examination of the factorial and convergent validity of four measures of conspiracist ideation, with recommendations for researchers. *PLOS ONE*, *12*(2).
