# Supplementary material for: Is there anything good about conspiracy beliefs? Belief in COVID-19 conspiracy theories is associated with benefits to well-being
Source: PLoS One. 2025 Mar 21;20(3):e0319896. doi: 10.1371/journal.pone.0319896 (PMC11927887; doi:10.1371/journal.pone.0319896)
Supplement: Attachment — Submitted filename: Response to Reviewers_Jan29.docx [file pone.0319896.s005.docx]

**Response to reviewers**

We would like to thank the reviewer for their comments on our manuscript and the editor for allowing us to revise the manuscript. We have carefully considered each comment, and have made multiple revisions to the manuscript to address the relevant concerns. We reproduce each comment offered in the action letter below in blue font, and provide responses in this black font. All page numbers referenced correspond to the “Manuscript with Track Changes” document.

**Editor:**

After careful consideration, we feel that your manuscript could be published but at this point does not fully satisfy R2. I believe these comments could be quickly addressed in a final revision after which I will make an in-house decision.

Thank you for allowing us to revise the manuscript. We have carefully considered each comment and made changes to the manuscript we believe makes it suitable for publication.

**Reviewer #2:**

1. Some of the causal claims have been modified in an appropriate fashion. However, in relation to the excerpts I highlighted in points 2, 3 and 5, the authors are still making causal inferences (“effect”, “benefits”, “boosts”), and without acknowledging the relevant uncertainties and/or assumptions. Even little cross-references like “but see the limitations subsection for the assumptions required for this interpretation…” could have done the trick.

We thank Reviewer 2 for encouraging us to be more explicit about our use of terms throughout the manuscript. We have elected to use causal language when describing our conceptual reasoning and hypotheses, but use language consistent with associations when describing our evidence. In support of the choice to use causal language in describing our hypotheses, we note that Reviewer 2 in their first review of the manuscript indicated that the “[t]he manuscript is very clearly motivated by questions about the causal effects of belief in specific conspiracy theories on wellbeing.” Despite the lack of experimental evidence, Reviewer 2 encouraged us to not completely back off making causal claims, but rather find additional methods by which to support causal inferences, which we did via propensity score weighting analyses and theoretical discussions of causal inference. Indeed, the reviewer stated:

What’s the solution to this? I should stress that I don’t think the answer is to back even further away from discussion of causal effects and present the paper as if it were only about associations. This paper is clearly motivated by questions about causal effects, and that’s totally ok. But I’d suggest really leaning into the topic of causal inferences, and how they can best be warranted and justified.

Thus, we believe that our choice to use causal language for hypotheses and associational language for results is well justified. We have inserted a paragraph on page 14 to clearly communicate our use of causal language to describe hypotheses and highlight our use of observational data to test them:

We have presented our conceptual reasoning in causal terms, highlighting the possibility that coming to believe a conspiracy theory about a specific event may benefit individuals’ well-being. However, we wish to acknowledge from the very outset that any such causal inferences ultimately require experimental evidence. The studies we present are correlational in nature. Although we will report a number of analyses aimed at buttressing the proposed hypothesis, it should be kept in mind that the findings are merely consistent with our conceptual framework. Nevertheless, we hope that the findings will inspire further research that offers converging evidence for causal inferences.

To ensure that our manuscript tightly adheres to this distinction in language, we have confirmed that results are either described as only associations or any language that may be construed as causal in nature is qualified by statements noting the lack of causal evidence. Examples of such edits can be found on pages 2, 22, 23, 40, 41, and 48.

As an example of edits involving removing causal language, we have edited the discussion after Study 1 on page 22:

The results of Study 1 provide initial support for our hypothesis: when people’s level of experienced turmoil and conspiracist ideation are taken into account, greater endorsement of specific conspiracy theories predicts less stress. That is, for two people who experienced the same level of economic turmoil and have the same level of conspiracist ideation, an increase in belief in COVID-19 conspiracy theories is associated with a decrease in stress. This suggests that believing conspiracy theories about a specific event can indeed be associated with psychological benefits for the believer, but uncovering this relation requires that researchers statistically separate the influence of experiencing turmoil and a general tendency to believe conspiracy theories from the belief in a conspiracy theory regarding the specific event. To our knowledge, this is the first instance in which a positive relation between believing conspiracy theories and stress has been documented.

Importantly, the evidence linking belief in an event conspiracy theory to reduced stress is strong from a statistical standpoint. Not only do the results reveal the predicted effect in a relatively large sample, the statistical evidence for the effect (*p* < 0.001) is impressive relative to even the most conservative standards [62]. However, a key limitation of Study 1 is the cross-sectional nature of the data, which seriously limits the ability to draw inferences about prospective relations among the variables. We address this limitation in Study 2.

Moreover, we also edited the text to include qualifying statements like that suggested by Reviewer 2:

Most importantly, this study provides evidence that believing in specific conspiracy theories *can* be associated with boosts in well-being (but see the limitations subsection for the assumptions required for this interpretation in the Discussion section) and clarifies that such relations are specific to increased contentment for the believer. (p. 40)

2. In my point 7, I asked for the “paragraph about causal limitations in the discussion section expanded into something a bit more comprehensive. What assumptions do readers need to make to interpret your beta values as unbiased estimates of causal effects? How realistic are those assumptions? What would happen if they're breached? E.g., one such assumption is that aside from conspiracist ideation and turmoil there exists no other third variable that affects both belief in specific conspiracy theories and wellbeing...” The authors have improved this paragraph, but not listed all these assumptions (just one that I happened to use as an example). What about the assumptions that the control variables are not colliders or mediators? To help them produce a more sophisticated description I’d suggest reading contemporary literature on causal inference (e.g. Rohrer, 2018, 2024). Thinking about simple concepts like covariance, temporal precedence and excluding alternative explanations is helpful when introducing students to causal inference, but at this level a bit more depth and specificity is called for.

We elected to focus on the inclusion of confounding variables in our discussion of assumptions required to interpret our results as unbiased estimates because it is the first and most extensively discussed issue in Rohrer (2018). Although we do not view an exhaustive list of such assumptions as being within the purview of our manuscript, we have expanded our discussion of the assumptions needed to interpret our results as unbiased estimates. Specifically, we now discuss two additional assumptions needed to interpret observational data as unbiased estimates of causal effects—accounting for error and not including mediator and collider variables. Thus, the revised paragraph lists three key assumptions according to Rohrer (2018), discusses the influence of failure to meet such assumptions, and evaluates them in relation to our data:

Before discussing the broader implications of this work, we wish to highlight three methodological aspects of this work we regard as important. First, given the lack of experimental evidence, strong causal claims are unwarranted. Establishing causality requires demonstrating evidence of covariation, temporal precedence, as well as ruling out plausible alternative explanations [70]. Across two studies, we provide evidence of covariation between event conspiracy beliefs and well-being. Although not definitive, the prospective nature of Study 2 provides some evidence regarding temporal precedence of the proposed relation between event conspiracy beliefs and well-being. Importantly, interpreting our results as unbiased estimates of the causal effect of event conspiracy beliefs on well-being relies on meeting a number of different assumptions. Naturally, a failure to rule out potential third variables would bias the estimates of the relation between event conspiracy beliefs and well-being, increasing the possibility of a spurious correlation. Although this research cannot definitively rule out all alternative explanations, it does leverage multiple regression and propensity score weighting analyses to rule out the most relevant situational, dispositional, and demographic variables as competing explanations based on the current state of the literature. In so doing, we furnish evidence that is consistent with the idea that believing specific conspiracy theories can have benefits for well-being. While it is possible that confounders other than those tested exist, we are unaware of likely candidates for the role. Moreover, the interpretation of results as unbiased estimates of a causal effect also relies on the degree of measurement error in confounding variables [71]. A failure to account for measurement error can lead to inflated Type I error rates [72]. One solution to this issue is the use of structural equation models (SEM), a method that estimates measurement error for multi-item measures. As a robustness check, we replicated our multiple regression analyses using SEM and found that our results remained substantively unchanged (see Supplemental Material). Although these results provide another converging line of evidence supporting our conclusions, our single-item measures of economic turmoil preclude us from modeling measurement error in this regard. Despite finding robust associations between economic turmoil and well-being, one valuable aspect of a future replication attempt would be the measurement of economic turmoil using a multi-item measure to ensure that this construct is assessed more comprehensively and measurement error can be modeled. Lastly, one additional assumption that enables an interpretation of our results as unbiased estimates of the causal effect involves is that the statistical models employed do not control for mediator or collider variables (see [71] for a full treatment of the assumptions that underlie interpreting observational data as an estimate of a causal effect). Inclusion of such variables in a model can either obscure or change the association between predictor and criterion variables. We provide a comprehensive explanation of our rationale for the causal ordering of variables in the introduction, which suggests that the confounders in our models do not act as either mediators or colliders. Nevertheless, it should be kept in mind that the current findings represent merely a step, albeit an important one in our view, in building a case for a causal relation. The research presented here establishes a positive association between event conspiracy beliefs and well-being. (pages 43-44)

3. Regarding my point 6: “the fact remains that your control variables all inevitably have at least *some* measurement error attached, your regression models assume they don't. Even without any other confounds this would be enough to cause the estimates of the focal causal effects to be biased (see Westfall & Yarkoni, 2016). I do think this needs to be acknowledged in the manuscript.” The authors respond by pointing to simulation by Gelman and Loken (2017). That simulation is interesting but of limited relevance to the current study: It’s a simulation relating to a bivariate relationship, not one where with control variables measured with error. Westfall and Yarkoni show that measurement error in covariates can cause biased estimates, and large sample sizes do not resolve this problem (in some cases they can even make it worse). The authors thus haven’t yet addressed my request.

We appreciate the opportunity to be clearer about our treatment of measurement error. First, we have expanded our discussion of measurement error in confounding variables and their impact on statistical models. Second, we note that Westfall and Yarkoni (2016) suggest the use of structural equation modeling (SEM) as a solution to controlling the inflated Type I error rate present in multiple regression models and point readers to the Supplemental Material, in which we present the results of SEM analyses on our data. These SEM models provide evidence consistent with our regression models. Thus, measurement error in confounding variables does not alter the substantive conclusions of our work. Importantly, however, our use of single-item measures of economic turmoil does not allow the SEM models to account for measurement error for this variable. As mentioned above with respect to the previous point, we address this issue by noting that we obtain robust relations between this variable and the well-known correlate of well-being, which suggests that there is not a great degree of error in our measure of economic turmoil. Notably, we conclude by suggesting that future research should use multi-item measures of economic turmoil to explicitly model measurement error:

Moreover, the interpretation of results as unbiased estimates of a causal effect also relies on the degree of measurement error in confounding variables [71]. A failure to account for measurement error can lead to inflated Type I error rates [72]. One solution to this issue is the use of structural equation models (SEM), a method that estimates measurement error for multi-item measures. As a robustness check, we replicated our multiple regression analyses using SEM and found that our results remained substantively unchanged (see Supplemental Material). Although these results provide another converging line of evidence supporting our conclusions, our single-item measures of economic turmoil preclude us from modeling measurement error in this regard. Despite finding robust associations between economic turmoil and well-being, one valuable aspect of a future replication attempt would be the measurement of economic turmoil using a multi-item measure to ensure that this construct is assessed more comprehensively and measurement error can be modeled. (pages 43-44)

**References**

Rohrer, J. M. (2018). Thinking clearly about correlations and causation: Graphical causal models for observational data. *Advances in methods and practices in psychological science*, *1*(1), 27-42.
