# Supplementary material for: Is there anything good about conspiracy beliefs? Belief in COVID-19 conspiracy theories is associated with benefits to well-being
Source: PLoS One. 2025 Mar 21;20(3):e0319896. doi: 10.1371/journal.pone.0319896 (PMC11927887; doi:10.1371/journal.pone.0319896)
Supplement: Attachment — Submitted filename: Response to reviewers.docx [file pone.0319896.s003.docx]

**Response to reviewers**

We would like to thank the editor and reviewers for their favorable reactions to our manuscript and for taking the time to provide helpful comments. We have carefully considered each comment, and have made multiple revisions to the manuscript. Our manuscript has greatly benefited from these revisions. We reproduce each comment offered in the action letter below in blue font, and provide responses in this black font. All page numbers referenced correspond to the “Manuscript with Track Changes” document.

**Editor:**

The comments from all three reviewers are quite constructive and could be addressed with thorough revisions. R1 praises the manuscript for its significant contribution to understanding the effects of conspiracy theory beliefs on well-being, suggesting minor revisions to strengthen the argument about the benefits of such beliefs. R2 and R3 both raise concerns about the manuscript's causal claims from correlational data, proposing a more rigorous justification for the causal model(s), clearer theoretical definitions, and some additional analyses to support the conclusions.

Thank you for this helpful summary. As we detail below, we have gone to great lengths to respond to the comments raised by the reviewers, including strengthening the arguments for why event conspiracy beliefs should have benefits for well-being, detailing the causal structure of the theoretical framework underpinning our work, clarifying definitions, and introducing new analyses to bolster causal claims.

**Reviewer #1:**

1. I think the authors’ argument regarding why belief in specific CTs can have benefits for individuals’ level of stress and well-being could be stronger. As it stands, there are only a few sentences about this (in the first paragraph under the “Current Research” heading and on the top of pg 10: “We contend that in the face of the kind of turmoil created by the COVID-19 pandemic…”) I recommend that the authors build this up a bit more, as this is the core of their argument – that belief in specific CTs can, in fact, be beneficial to individuals’ well-being (even if the benefits don’t last all that long).

We thank the reviewer for the encouragement to bolster the rationale for our predictions. We have inserted text on page 13 to better articulate why belief in a specific conspiracy theory can provide benefits for one’s level of stress and well-being:

“The need to account for the experience of turmoil when examining potential benefits of event conspiracy beliefs seems relatively straightforward. However, readers may wonder why we propose that an event conspiracy belief can have benefits for people’s level of stress and well-being, while the general tendency to believe conspiracy theories does not. We contend that, during a period of uncertainty and confusion like that created by the COVID-19 pandemic, individuals who endorse event-related conspiracy theories—logically flawed as they may sometimes be (Albarracin et al., 2021)—may fare better than those left without a clear causal explanation (Giacomantonio et al., 2022). That is, non-conspiracy explanations, such as random chance or human error, mean the world is unpredictable, which is itself stressful (Grupe & Nitschke, 2013). When a group of people is conspiring, the world may seem less unpredictable. Moreover, such non-conspiracy explanations may feel insufficient or trivial in the face of a global crisis due to proportionality bias—the tendency to believe that large effects stem from large causes (Bortolotti, 2023). Thus, a global shutdown may be more easily understood as being caused by the evildoings of global elite than by a virus spread in a wet market.

Conversely, higher levels of conspiracist ideation are unlikely to furnish such an overall benefit to the believer. The frequent tendency to see dark forces operating in numerous domains of one's life and the perseveration on those forces that accompany higher levels of conspiracist ideation is obviously stressful, as noted earlier (e.g., Freeman & Bentall, 2017).”

2. I’m not sure it’s necessary to show the impact of specific CT beliefs while separately controlling for conspiratorial thinking and turmoil, then showing the effects while controlling for both conspiratorial thinking and turmoil in the same model. The authors aren’t interested in teasing apart the relative impact of the two variables (conspiratorial thinking or turmoil). As such, I think the authors can streamline the presentation of their results by focusing only on the models controlling for both variables. The other models could be moved to an appendix.

We appreciate the reviewer’s perspective and carefully deliberated regarding this issue. Although we see the value in streamlining the presentation of the analyses, we have elected to retain the original presentation of the results because the more thorough presentation of our findings highlights the importance of each variable in its own right for the reader.

3. With regard to the relationship between conspiratorial thinking and (poorer) well-being, the authors could also site Farhart et al. (2021):

Thank you for bringing our attention to this citation. We have included this citation in the text on pages 9 and 11.

**Reviewer #2:**

Despite these positive points, I have one core (and relatively substantial) concern about the manuscript, and it relates to the topic of causal inference. The manuscript is very clearly motivated by questions about the causal effects of belief in specific conspiracy theories on wellbeing. For example, the authors say, “The central thesis guiding the current research is that believing specific conspiracy theories can have benefits for the believer, although such benefits may diminish over time.” To produce a benefit is to have a causal effect. The abstract and discussion also make anumber of implicit causal inferences, e.g., “The current research provides evidence for benefits of endorsing specific conspiracy theories”; “conspiracy beliefs do provide at least temporary intrapersonal benefits”; “Across two studies, one cross-sectional in nature and the other prospective, we find converging evidence that believing specific conspiracy theories can have psychological benefits for the believer.” Yet the data is all purely observational/correlational. The authors acknowledge this by saying “given the correlational nature of this research, strong causal claims are unwarranted”, yet the manuscript does make strong causal claims (indeed, there’s one in the title). This presents a problem in terms of the relationship between the evidence presented and the claims made.

Obviously Study 2 is longitudinal, but longitudinal data isn’t a magic bullet for causal inference; its capacity to support causal claims depends on the nature of the data and the analyses conducted. E.g., some specific longitudinal designs and analyses (e.g., interrupted time series designs, random intercept cross lagged panel models) can permit at least some types of alternative explanations to be ruled out. But that isn’t the case here, where the analyses are just regression models include variables from different waves. Substantively, any number of third/confounding variables could affect both belief in conspiracy theories at one point in time and wellbeing at another point in time, and this study only has a strategy to control for two of them (turmoil, conspiracist ideation). The longitudinal analyses presented here therefore don’t provide much stronger evidence of causality than the cross-sectional ones.

What’s the solution to this? I should stress that I don’t think the answer is to back even further away from discussion of causal effects and present the paper as if it were only about associations. This paper is clearly motivated by questions about causal effects, and that’s totally ok. But I’d suggest really leaning into the topic of causal inferences, and how they can best be warranted and justified. Specifically:

1. Provide strong causal justifications for the variables controlled (and the ones you chose not to control). See Wysocki et al. (2022).
2. Be explicit about the fact that the paper is attempting to identify causal effects, and the assumptions required to interpret the estimates as unbiased estimates of causal effects (see Grosz et al., 2020; Rohrer, 2024).
3. Consider whether there are alternative data sources or analyses you could specify here that would provide stronger evidence of causal effects (see Rohrer, 2018). One possibility might be an additional longitudinal study, designed specifically for this paper, analysed via the random intercepts cross lagged panel model (Hamaker et al., 2015). Doing so might open up the opportunity to preregister analyses and subject the hypotheses to a severe test (see Lakens, 2019). Obviously this is a big ask in a peer review, and I’ll leave it up to the authors and editor to decide whether this is appropriate.

We very much agree that the theoretical framework driving our research proposes a causal relation between belief in event conspiracy theories and well-being. Given the correlational nature of the data, however, the original submission strived to not make strong claims regarding causality. We do appreciate the encouragement to seek alternatives to enable stronger claims regarding causality. As suggested, we have taken a number of steps to enable stronger causal inferences.

First, we have followed Reviewer 2’s advice by bolstering the existing justification for the variables included in our framework and explained why we did not include other variables that may seem relevant. On pages 10 and 11, we write:

“We elected to focus on these variables *a priori* because they are the most salient situational and dispositional factors in the development of event conspiracy beliefs in current research and theory in the field (e.g., Douglas et al., 2017; Swami et al., 2017).

We elected not to include additional potentially relevant variables like political ideology and demographic characteristics because they seemed less able to account for the key relation of interest—that between an event conspiracy belief and well-being. For example, some prior work suggests that greater political extremity predicts greater belief in conspiracy theories (Imhoff et al., 2022), whereas other work suggests no systematic relation beyond greater belief in ideologically-congruent conspiracy theories (Uscinski et al., 2022). Moreover, although age, education, income, and race are consistently linked to belief in conspiracy theories, these associations are, at best, modest (Enders et al., 2024). In support of this choice, exploratory analyses that introduce political ideology and demographic variables into the relevant statistical models render the results virtually unchanged (see Supplemental Material).

The theoretical framework guiding this research postulates that believing in an event conspiracy theory directly causes an increase in well-being—at least momentarily—but both the experience of turmoil and conspiracist ideation exert causal effects on both well-being and belief in an event conspiracy theory, with the result being a suppression of the positive relation between event conspiracy beliefs and well-being (see Figure 1).

In support of the hypothesized beneficial effects of believing event conspiracy theories on well-being, we outline the evidence for other paths in the model below. First, there is evidence that both experiencing turmoil and conspiracist ideation promote belief in a given event conspiracy belief. Specifically, experiments have established a causal link between the experience of turmoil and distress to endorsement of more specific conspiracy theories (Casara et al., 2022; Grzesiak-Feldman, 2013). Although experimental evidence is hard to come by given the practical and ethical concerns, conspiracist ideation robustly predicts more specific event conspiracy theories (e.g., Brotherton et al., 2013; Swami et al., 2017), which suggests that the general tendency to believe conspiracy theories leads people to be more likely to interpret explanations for a given event in a more conspiratorial manner. Moreover, both the experience of turmoil and conspiracist ideation are themselves also associated with greater stress and poorer well-being. Not surprisingly, experiencing economic turmoil is associated with decreases in well-being (e.g., Bayliss et al., 2017; Park et al., 2017; Stallings et al., 1997; Tausig & Fenwick, 1999). Similarly, conspiracist ideation has been linked to indicators of poorer well-being (e.g., Farhart et al., 2021; Freeman & Bentall, 2017; Poon et al., 2023), although there are some conflicting findings in the literature (Georgiou et al., 2020). Thus, the predicted positive relation between belief in event conspiracy theories and well-being may be obscured by the dual influence of turmoil and conspiracist ideation in increasing event conspiracy beliefs and lowering well-being, resulting in simple relations that are either null (Liekefett et al., 2021) or negative (van Prooijen et al., 2021).

Second, we have clearly stated that our framework postulates causal effects:

“The theoretical framework guiding this research postulates that believing in an event conspiracy theory directly causes an increase in well-being—at least momentarily—but both the experience of turmoil and conspiracist ideation exert causal effects on both well-being and belief in an event conspiracy theory, with the result being a suppression of the positive relation between event conspiracy beliefs and well-being (see Figure 1).” (pgs. 10-11)

Moreover, we explain the bounds of our inferences given the lack of experimental data while noting the ways in which the analyses we present do fit the criteria for establishing causality:

“First, given the lack of experimental evidence, strong causal claims are unwarranted. Although this research cannot rule out all alternative explanations, it does leverage multiple regression and propensity score weighting analyses to furnish evidence that is consistent with the idea that believing specific conspiracy theories can have benefits for well-being. In this way, the findings represent the important step of building a case for a causal relation by establishing an association between event conspiracy beliefs and well-being.” (p. 43)

Lastly, we carefully considered how to better support the claims made in the manuscript. Unfortunately, the cost of conducting an additional longitudinal study is prohibitive in terms of both resources and time and we were unable to find alternative openly available data sources that contained the variables necessary to test our hypotheses. However, we used new analyses of our existing data to bolster our conclusions regarding the effect of belief in COVID-19 conspiracy theories on well-being. First, even though political ideology and demographic variables were not a part of our theoretical framework, we decided to include them in an additional step of our multiple regression analyses to ensure that they did not account for the significant relations reported in the text. To not overwhelm the reader, we report the key result of these analyses in the main text and refer interested readers to the Supplemental Material for more details. For Study 1, we write:

“We conducted an additional analysis to ensure the robustness of our results. Although not a part of our theoretical framework, we included political ideology, age, race, income, and education as additional covariates in a multiple regression analysis including all prior predictors. Even when controlling for political ideology and demographics in addition to turmoil and conspiracist ideation, belief in COVID-19 conspiracy theories continued to predict lower levels of stress (see Supplemental Material for details).” (pgs. 21)

For Study 2, we write:

“As in Study 1, we checked the robustness of our results by including political ideology and demographic variables (age, race, income, and education) into the regression models discussed above. The inclusion of these additional covariates resulted in no changes to the substantive interpretation of the results. In particular, belief in COVID-19 conspiracy theories continued to predict greater contentment at Wave 3 even when statistically accounting for the experience of turmoil, conspiracist ideation, political ideology, and demographic characteristics.” (pgs. 37-38)

Second, we used propensity score weighting to provide independent tests of the hypothesis that belief in COVID-19 conspiracy theories provided benefits to well-being. Importantly, we also included a brief description of the advantage that this method provides:

“Given our interest in testing whether event conspiracy beliefs exert a causal effect on stress, we performed an exploratory analysis using the propensity score balancing technique with stress as the outcome, belief in COVID-19 conspiracy theories as a continuous treatment variable, and our key covariates—the experience of economic turmoil and conspiracist ideation—as confounders. Propensity score weighting tries to mimic characteristics of a randomized control trial by balancing the distribution of potential confounders across values of the focal predictor (Fong et al., 2018).” (pgs. 21-22)

We report the results of the propensity score weighting analyses for Study 1 on pages 21 and 22 and Study 2 on pages 38 and 39. Moreover, to ensure that these findings were not dependent on our decision to focus on the confounders in our theoretical framework, we conducted additional analyses in which we also included political ideology and demographic characteristics that have been implicated in conspiracy beliefs and well-being. Those results are reported on pages 13 to 17 of the Supplemental Material.

MINOR POINTS
4. The paper reports a lot of analyses. There are a bunch of different operationalisations of the dependent variables, different lags, models with each combination of the control variables, etc. Of course I would much rather the authors did that then cherrypick only a subset to report, but one problem resulting from the number of models and lack of a preregistration is that there are multiple reasonable interpretations of the results one could draw, considering they’re quite a mix of significant and non-significant coefficients for beliefs in conspiracy theories. The authors see a pattern that there is some effect of belief in conspiracy theories on wellbeing in the short term that disappears in the longer term (hence the non-significant effects at longer lags). This is one reasonable interpretation, but the differing findings at different lags could just as well be interpreted as suggesting there’s a fair bit of noise present and the evidence of effects is tentative and inconsistent. As a reader it’s consequently rather hard to tell to what extent the hypotheses faced a genuine risk of falsification. This is another reason why I think that another, preregistered, longitudinal study would really help here – albeit again I know this is a big ask.

We agree that many analyses are presented in the manuscript. We also believe, however, that the data present a very clear picture. In Study 1, belief in COVID-19 conspiracy theories predict less stress when controlling for either only the experience of turmoil, only conspiracist ideation, or both together. These results are functionally the same when including political ideology and demographic characteristics as additional covariates. Moreover, this pattern of results is paralleled by the propensity score weighting analyses in which belief in COVID-19 conspiracy theories predict stress when using (1) the experience of turmoil and conspiracist ideation as confounders and when (2) adding political ideology and demographic characteristics as confounders.

“When looking across analyses, then, there is a consistent relation between belief in COVID-19 conspiracy theories and stress once economic turmoil and conspiracist ideation are taken into account. (pg. 22)

In Study 2, when accounting for economic turmoil and conspiracist ideation, belief in COVID-19 conspiracy theories only predict contentment at Wave 3, but not contentment at Wave 5 or negative symptomology at either Wave 3 or Wave 5. Moreover, these results are obtained using either multiple regression or propensity score weighting analyses and independent of whether only the experience of economic turmoil and conspiracist ideation are included in the model or whether political ideology and demographic characteristics are also included. Thus, the beneficial effects of believing event conspiracy theories are apparent once the key covariates—the experience of economic turmoil and conspiracist ideation—are included in the relevant model and this effect appear to be time-sensitive, as indicated by a significant interaction between belief in COVID-19 conspiracy theories and time in a mixed model reported on page 38. We have inserted text on page 39 to communicate this overview:

“Looking across analyses, a clear picture emerges: belief in COVID-19 conspiracy theories predicts greater contentment in the short-term, but this effect dissipates over time. No relation emerges between belief in COVID-19 conspiracy theories and negative symptomology. Importantly, these findings hold when using both multiple linear regression and propensity score weighting and when using only economic turmoil and conspiracist ideation as covariates or using these covariates in addition to political ideology and demographic characteristics.”

5. P. 7, “Douglas and colleagues (2019) found that both belief in conspiracy theories regarding a given event or issue (e.g., 9/11 or genetically modified foods) and conspiracist ideation have primarily negative consequences”. The empirical findings cited in the paragraphs that follow here seem to be mainly correlational (‘consequences’ implies causal effects).

Thank you for bringing our attention to this matter. We have addressed this issue in two ways. First, we have softened the causal language given that we discuss a mixture of correlational and experimental findings:

“Douglas and colleagues (2019) found that both belief in conspiracy theories regarding a given event or issue (e.g., 9/11 or genetically modified foods) and conspiracist ideation are primarily associated with undesirable outcomes.” (pg. 7)

Moreover, we have added an example of experimental evidence linking exposure to specific conspiracy theories to undesirable outcomes:

“Experimental evidence suggests that exposure to anti-vaccine conspiracy theories lowers vaccination intentions (Jolley & Douglas, 2014a).” (pg. 7)

The literature on conspiracist ideation is almost exclusively correlational. We are not aware of any experiments testing whether experimentally-created increases in conspiracist ideation influence well-being, and thus, represents the best available evidence regarding its consequences.

6. Economic turmoil is a key control variable in Study 1, but is measured via just a single and relatively narrow item. If this item contains measurement error as a measure of turmoil (which will be the case), statistically controlling for it in a regression model doesn’t fully control for the effects of turmoil (see Westfall & Yarkoni, 2016). Similar issues, albeit less serious, apply to the other control measures.

Thank you for bringing attention to this issue. We agree that single item measures can sometimes be problematic. For instance, when there is a desire to measure a broad, multi-faceted construct like personality, single-item measures may not be ideal. However, single-item measures are not necessarily inferior to multi-item measures (Allen et al., 2022). The construct of interest in our research was the degree to which people experienced economic turmoil as a result of the COVID-19 pandemic. Although one may construct a multi-item measure of this construct by asking many questions about a person’s financial situation, such questions may not capture the true impact of the pandemic given that it depends on the interplay between losses to income, savings, family support, etc. Moreover, a principle of social psychology is that people’s subjective construal of a situation shapes people’s thoughts, feelings, and behavior. Lastly, there is no reason to believe that a single-item measure of the economic turmoil cannot capture people’s general evaluation of how the pandemic impacted their finances. Thus, we contend that the single-item measures used to capture economic turmoil are valid from a theoretical perspective because they tap into people’s subjective construals of how the pandemic impacted their financial situation.

Regarding the issue of measurement error, some maintain that single-item measures are necessarily inferior to multi-item measures. Although there is debate about the validity of this statement (Allen et al., 2022), an examination of the results suggests that even if the single-item measures of economic turmoil used in this research are noisier, they contain sufficient signal to allow meaningful analysis. Specifically, the single-item measures of economic turmoil are robust predictors of stress (Study 1) and both aspects of well-being (negative symptomology and contentment; Study 2), a pattern that replicates prior findings in the literature (Bayliss et al., 2017; Park et al., 2017; Stallings et al., 1997; Tausig & Fenwick, 1999).

7. Given the key role of the measure of stress as a dependent variable in Study 1, it’s likewise a bit surprising that it was measured via just a single ad hoc item. Why not use a validated measure of stress? The measurement error resulting from this decision won’t bias the results per se (since it’s measurement error in the DV), but will add noise and reduce power.

We appreciate the opportunity to discuss the rationale for using a single-item measure of stress and discuss its merits. As discussed in the original submission, this research involves large datasets that were collected during the COVID-19 pandemic with multiple aims in mind. The constraints of conducting large surveys in a manner that does not overload participants imposed time constraints, the result of which was the use of briefer measures of constructs of interest. As such, the source data for Study 1 measures stress via a single-item measure instead of a multi-item instrument. If we assume that our measure of stress is noisier than an alternative multi-item instrument, the reduction in power makes our findings all the more remarkable given that we replicate known relations (see above) and extend the literature by showing a beneficial effect of belief in an event conspiracy theory.

8. I’m not sure how much value there is in the factor analysis in of conspiracism items in Study 1. Given that the authors specified a 2-factor model without using a robust method for selecting the number of factors (c.f. the methods in Hayton et al., 2004; Zwick & Velicer, 1986), this can’t really be interpreted as a test of the idea that the factors “represent two related but distinct constructs.”

Thank you for bringing our attention to parallel analysis as a method for selecting the number of factors in a factor analysis. Using the *psych* package in R, we conducted a parallel analysis to determine the number of factors in the relevant data. Consistent with our theoretically-derived predictions, there are two factors in the data. We have inserted text on page 19:

“To this end, we first used parallel analysis (Hayton et al., 2004) to determine the number of factors in these conspiracy item-related data. The results revealed two factors in the data. We followed this analysis by estimating a model using principal axis factoring with a direct oblimin (oblique) rotation, and requested the extraction of two factors.”

The relevant syntax can be found on our OSF page (https://osf.io/7b3xp/?view_only=f649bb67327c40629d9b63b3b2188fd5).

9. I take it the beta values reported are all standardized? It’d be good to make this clear at first use.

That is correct. Beta values represent standardized regression coefficients. We note this in the original submission in relation to Study 1 on page 20:

“All variables were standardized (z-scored) prior to analysis.”

As we will discuss later, we have added information that conveys we z-scored the variables (as opposed to mean-centering) given Reviewer 3’s questions on this matter.

We have added a parallel sentence to indicate that all beta values represent standardized regression coefficients in Study 2 on page 34.

10. The interpretation that the “the benefits with respect to contentment that were evident at Wave 3 had dissipated substantially nine months later” isn’t strongly warranted by the findings. This claim implicitly suggests the effects were smaller at longer lags, but the models reported don’t test whether they were significantly smaller. The fact that they were mostly significant at short lags and not at long lags isn’t in of itself evidence of a significant difference in effects over time; “The difference between “significant” and “not significant” is not itself statistically significant” (Gelman & Stern, 2006). To test the idea that the effects dissipated over time would require an analysis specifically targeted to this question, likely involving some kind of interaction term.

We are very much in agreement that the difference between a statistically significant regression coefficient and one that is not statistically significant is not indicative of a significant difference between the two. Accordingly, the original submission presented the results of a mixed model in which we tested for this difference via an interaction between time on page 35. To better highlight this analysis, we have added the heading “Mixed Model Comparing Regression Coefficients across Time” on what is now page 38.

11. I’m not convinced that “Coronavirus is actually no more dangerous than the common flu” is a conspiracy theory; it doesn’t allege a conspiracy. I can see the authors anticipated this objection and ran a small empirical study to show that people tend to think beliefs in this item would be associated with belief in conspiracy theories, but that’s not evidence of content validity per se. It might be useful to highlight in the main text how the main findings hold up when only the other, more clearly conspiratorial, item is treated as the dependent variable. (I think there might be some analyses of this in the supplementary materials but haven’t checked those in detail - apologies).

We maintain that the data we present on page 26, which indicate that people interpreted these items as being conspiratorial, support our assertion that the items in Study 2 do indeed tap into conspiracy beliefs about the COVID-19 pandemic. Nevertheless, we do present analyses in the Supplemental Material in which we show that each item of our COVID-19 conspiracy belief composite in Study 2 significantly predicts contentment at Wave 3. Although the original manuscript noted that the results of Study 2 remain substantively unchanged when using each individual COVID-19 conspiracy item in the Methods section, we now reiterate this statement in the Results section to make this finding clearer to the reader:

“The results remain substantively unchanged when using each item assessing COVID-19 conspiracy beliefs as a predictor instead of the composite measure (see Supplemental Material for full details).” (p. 35)

12. I don’t know that the use of factor scores for dependent variables in Study 2 was the optimal strategy. I appreciate why the authors did this, but the problem of factor score indeterminacy looms here (see Grice, 2001). There is an infinite number of different factor scores that can be computed from the same factor solution, and these different sets of factor scores would each produce different substantive findings. Would it not be a better idea to use structural equation modelling and treat the variables as latent?

We understand the concern regarding our decision to model our outcomes in Study 2 using factor scores. To alleviate this concern, we used SEM to model well-being using latent variables. Before proceeding to the results of SEM analyses involving Study 2, we note that the results of Study 1 remain substantively unchanged when using SEM instead of multiple linear regression.

As for Study 2, we used the *lavaan* package in R to estimate SEM models in which well-being was modeled using two latent variables—one for negative symptomology and one for contentment. Although not presented in the manuscript due to the number of analyses already presented, the results of the SEM models support the findings reported in the in the main text. Specifically, belief in COVID-19 conspiracy theories continues to predict contentment at Wave 3 when economic turmoil and conspiracist ideation are in the model—a pattern that is absent in the Wave 5 data. Moreover, this difference in the strength of the relation between COVID-19 conspiracy beliefs and contentment is statistically significant, as indicated by a test of the difference between a model in which the coefficient for belief in COVID-19 conspiracy theories is allowed to vary between Wave 3 and Wave 5 and a model in which it is not. These findings are reported on pages 13 to 14 of the Supplemental Material. The syntax associated with these analyses can be accessed via our OSF page (https://osf.io/7b3xp/?view_only=f649bb67327c40629d9b63b3b2188fd5).

13. Relatedly, the criteria on which the authors base the conclusion that the wave 3 wellbeing responses produced a 2-factor solution could have been clearer

Thank you for pointing to the lack of clarity regarding the factor analysis reported for Wave 3 data in Study 2. The original identification of the two factors of well-being in Study 2 were based on the Kaiser criterion and an inspection of the scree plot, both of which suggested a two-factor solution. This was consistent with our *a priori* expectations: according to prominent theoretical perspectives, well-being can be understood in terms of positive and negative affect (Diener, 1984; Diener et al., 2002).

14. Re. the control for conspiracist ideation, I can understand the authors’ rationale on this, but it’s hard to rule out the possibility that belief in specific conspiracy theories itself affects conspiracist ideation, which then goes on affect stress or wellbeing. Indeed, if I develop a belief in a specific conspiracy theory it’s not unreasonable to suppose this might make me more amenable to the idea that in general conspiracies happen frequently. If so, this would make conspiracist ideation a mediator and not just a confounding variable, such that controlling for it could add bias to the estimate of the effect of belief in specific theories. I don’t think this is a solvable problem per se, but do make the assumptions clear (e.g., to interpret the coefficients in the models with both controls as unbiased estimates of causal effects, we need to assume that belief in specific theories doesn’t affect either of the control variables).

This is an interesting point. First, we have added text to our figure caption on page 12 to clarify that our theoretical framework represents the events unfolding at one point in time. As such, the dynamics between belief in a specific conspiracy theory and conspiracist ideation are not modeled:

“To be clear, this representation depicts the proposed psychological processes occurring at one point in time. Although there is evidence that belief in COVID-19 conspiracy theories can lead to increases in conspiracist ideation (Granados Samayoa et al., 2022), the model limits itself to situations in which this recursive mechanism does not operate.”

Importantly, however, we do not regard the idea of conspiracist ideation acting as a mediator as a viable alternative explanation because a mediational model would posit that there is a positive indirect effect of belief in COVID-19 conspiracy theories on stress through conspiracist ideation. That is, greater belief in COVID-19 conspiracy theories would be associated with greater stress through the influence of such belief on conspiracist ideation. There is no evidence of such an indirect effect in our data, *ab* = .08 95% CI [-0.02, 0.19].

15. “although our work suggests that believing a specific conspiracy theory may make a person feel better in the moment, these effects disappear over time, and have very real dangers for them and those around them.” I don’t think this study really shows evidence of dangers of beliefs in conspiracy theories; perhaps make it clearer that this claim stems from other evidence sources.

Thank you for bringing our attention to this imprecise language. We have edited the text on page 45 to read:

“Indeed, although our work suggests that believing a specific event conspiracy theory may make a person feel better in the moment, these effects disappear over time. Importantly, a growing body of research suggests that conspiracy beliefs have very real dangers for believers and those around them (see Douglas et al., 2019, for a review).”

**Reviewer #3:**

1. Causal theory underpinning the aims and analyses.
At the core of the paper is the idea that specific conspiracy theory belief (CT) predicts improved mental health (MH) but only when we control for conspiracy ideation (CI) and/or economic turmoil (ET).

Across the two studies it is mostly found that, by itself, CT is associated with poorer MH or is not associated at all (consistent with previous research). When CI and ET are added as predictors (both by themselves and together) the relationship changes so that the estimate for CT is associated with improved MH. In other words, both CM and ET appear to be acting as suppressors. The question that needs answering is ‘why they are doing that?’ Is it possible that (1) the suppression is some kind of statistical artefact, or (2) does it suggest some deeper causal complexity that needs unpacking (e.g., a mediation model of some kind)?

My overriding question is ‘why do you want to control for CI and ET?’ - what kind of causal theory is motivating this? Why (and how), for instance, do we think that CI might be ‘hiding’ the true relationship between CT and MH? Both studies suggest that ‘that part of CT that is independent of CI and ET’ (call it CT-[CI+ET]) is positively related to MH. But what is CT-[CI+ET]? And what are the practical implications of this ‘thing’ being positively related to MH? At several points in the paper, it is implied that CT belief predicts better MH (as if CT-[CI+ET] is some kind of ‘purer measure’ of CT belief). Yet I don’t think this necessarily follows. Whatever CT-[CI+ET] is, it isn’t ‘CT simpliciter’. In fact, it seems to me to be a particularly strange kind of CT belief that is independent of the political and epistemic attitudes that underpin CI and independent of situational hardship drivers indexed by ET – it’s CT belief stripped of internal and situational mechanisms commonly associated with CT belief. It would be an understatement to say that I’m intrigued about the findings and would very much like to see some attempt to interpret the suppression effects.

Thank you for your comment. We have expanded our discussion of why the experience of economic turmoil and conspiracist ideation obscure the significant relation between belief in event conspiracy theories and well-being. As noted above, we have included a diagram that better represents the theoretical framework guiding this research on page 12. Moreover, we have provided a more detailed explanation of the causal relations between the experience of economic turmoil and conspiracist ideation and both event conspiracy beliefs and well-being on page 10 of the main text and in our response below Reviewer 2’s third comment. For the sake of brevity, we will not reproduce this text in its entirety. In brief, our research was motivated by the idea that the experience of economic turmoil and conspiracist ideation both lead to an increase in event conspiracy beliefs. Moreover, both economic turmoil and conspiracist ideation are associated with poorer well-being. Thus, these two covariates act as confounds when trying to assess the relation between belief in a specific conspiracy theory and well-being that pull the direction of this relation in the negative direction. When both are accounted for, we see benefits of event conspiracy beliefs on well-being.

Regarding the interpretation of our index of COVID-19 conspiracy beliefs in the context of our multiple regressions, we would like to start by noting that statistically controlling for covariates like the experience of economic turmoil and conspiracist ideation does not change the kind of belief captured by our index of COVID-19 conspiracy beliefs—they are still COVID-19 conspiracy beliefs. What our index of COVID-19 conspiracy beliefs represents when controlling for conspiracist ideation is the residual variance once we account for the general bias to believe conspiracy theories. As we detail in the paper, there are many reasons why people believe in conspiracy theories, and thus, plenty of variance is left over.

Moreover, we note that our theoretical framework proposes that some portion of the relation between COVID-19 conspiracy beliefs and well-being will be attributable to the influence of experiencing economic turmoil and some will be attributable to conspiracist ideation. Darlington and Hayes (2017) state that coefficients in a multiple regression “quantify the relationship between Y and each regressor while holding all other regressors constant” (p. 58). Thus, the multiple regression models in our paper sever the association between belief in COVID-19 conspiracy theories and both economic turmoil and conspiracist ideation. This means that we can examine the relation between belief in COVID-19 conspiracy theories and well-being when economic turmoil and conspiracist ideation do not change (i.e., when the focal association is not influenced by changes in economic turmoil and conspiracist ideation). So, one way to interpret our results that is less likely to lead to confusion is to say that for two people who have experienced the same amount of economic turmoil and have the same level of conspiracist ideation, an increase in endorsement of COVID-19 conspiracy theories during the COVID-19 pandemic was associated with an increase in well-being in the form of reduced stress and increased contentment. In this way, multiple regression offers an index of the contribution of COVID-19 conspiracy beliefs to well-being, independent of the influence of experiencing economic turmoil and conspiracist ideation. We have integrated this explanation into the text on page 44:

“More broadly, multiple regression allows us to obtain an estimate of the influence of COVID-19 conspiracy beliefs on well-being when holding the experience of economic turmoil and conspiracist ideation constant. In this way, it provides an index of the unique relation between COVID-19 conspiracy beliefs and well-being with the influence of economic turmoil and conspiracist ideation removed. One way to interpret our findings is that for two people who have experienced the same amount of economic turmoil and have the same level of conspiracist ideation, an increase in endorsement of COVID-19 conspiracy theories during the COVID-19 pandemic was associated with an increase in well-being in the form of reduced stress and increased contentment.”

We have also added expanded interpretations of the regression coefficients to the discussion section of each study:

“That is, for two people who experienced the same level of economic turmoil and have the same level of conspiracist ideation, an increase in belief in COVID-19 conspiracy theories is associated with a decrease in stress.” (pg. 22)

“One way to interpret this finding is that for two people who experienced the same level of economic turmoil and have the same level of conspiracist ideation, an increase in belief in COVID-19 conspiracy theories is associated with an increase in contentment.” (p. 40)

Here are a few suggested additions that might make some of the relevant issues clearer to readers:
• To address statistical questions readers might have, include zero-order correlations between predictors and predictors and DVs/criterion variables and measures of multicollinearity (e.g., VIF). Simple slopes analyses or interaction analyses might also go some way to helping people unpack what is going on when the CI and ET are added to models.

Thank you for these helpful suggestions. First, we have added correlation matrices to the manuscript. The correlation matrix for Study 1 can be found on page 19. The correlation matrix for Study 2 can be found on page 33. Second, we report that all VIF values are well below the conservative recommended threshold of 5 for both Studies 1 and 2:

“All variance inflation factor (VIF) values were well below commonly used thresholds for issues of multicollinearity (all VIFs < 2.4).” (pg. 20)

“All VIF values were well below commonly used thresholds for issues of multicollinearity (all VIFs < 1.2).” (pg. 34)

Although we appreciate the suggestion to conduct analyses involving interactions, we regard this question as being beyond the scope of the current research and its aims.

• Re-work the introduction to make it clearer why the CI and ET variables are being controlled or are being treated as ‘confounds’ of the CT-MH relationship. If the causal relationships that underpin the decisions to do the analyses in the two studies could be represented in a Directed Acyclic Graph (DAG) I think readers will be better able to grasp the authors’ theoretical assumptions.

Thank you for this suggestion. We have indeed expanded our discussion of the proposed causal relations underpinning our work and inserted a figure to depict these relations graphically in pages 9-12 of the main text, as more thoroughly discussed above.

2. Clarifying what is meant by specific conspiracy theory and conspiracy ideation.
There’s a lack of clarity in the introduction about the difference between conspiracy ideation/mentality and belief in specific conspiracy theories. At times it seems like the term ‘specific conspiracy theory’ is intended to capture belief in a single specific CT. Yet in both studies ‘specific conspiracy theory’ is measured by the combined ratings of TWO specific CTs (although it’s good to see that the supplementary materials also examine ‘lone’ specific CTs).

We appreciate being alerted to this potential point of confusion. Our use of the terms “belief in a specific conspiracy theory” and “conspiracist ideation” was guided by classic work in the conspiracy theory literature (Brotherton et al., 2013; Douglas et al., 2019; Swami et al., 2010; 2017). We agree that recent developments in the literature have made the use of the terms above less clear. We believe this to be a matter of conflating the measure with the construct. The fact that the BCTI uses real-world conspiracy theories and the GCBS uses generic statements does not mean they measure different constructs. However, this matter is beyond the scope of this project.

To steer clear of confusion created by recent developments in the use of the terms, however, we have changed our terminology when referencing out key predictor. Specifically, the construct represented by belief in COVID-19 conspiracy theories is now referred to as “an event conspiracy belief,” a term that builds on Barkun’s (2013) classification of types of conspiracy theories. We define event conspiracy beliefs as “subjective convictions regarding the existence of a conspiracy in relation to a specific event or set of events” (p. 5). We have made this change in terminology through the text. Moreover, we have significantly trimmed the section “Individual Differences in Belief in Conspiracy Theories” (pgs. 4-5) to only focus on measures of conspiracy mentality that use generic statements like the Generic Conspiracist Beliefs Scale and the Conspiracy Mentality Questionnaire.

On pages 4-5 it is suggested that Goertzel (1994) measured CI (“there was sufficient coherence in the data to create an index of conspiracist ideation”), yet he asked people about a collection of specific CTs (which isn’t the same thing as far as I can tell). The current paper seems to be using different definitions of specific CT and conspiracy ideation (where the average belief for a collection of specific conspiracy theories is considered to be a measure of CI) from that, that I’m used to.

With regard to the work of Goertzel (1994) specifically, he writes that part of the motivation for his work was the lack of research that had “addressed the question of to what extent belief in conspiracies is a generalized ideological trait” (p. 732). Indeed, he regards this as his primary aim: “our first goal was to what extent there is a generalized tendency to believe in conspiracies” (p. 735). Goertzel conducts a factor analysis of responses to the various real-world conspiracy items and finds that “the first principal factor explained 37.8% of the variance common to these items” (p. 735). On the basis of psychometric analyses, Goertzel concludes: “These findings offer strong support for the hypothesis that belief in conspiracies is a generalized ideological dimension” (p. 735).

Lastly, Goertzel constructs a scale reflecting the general tendency to believe conspiracy theories using the 10 items tapping into different real-world conspiracy theories:

“These 10 items were used to construct a summated scale of Belief in Conspiracies. This scale had a reliability coefficient (a) of .78, confirming that the items have enough variance in common to justify treating them as a scale for this population.” (p. 736)

I don’t think this is what Imhoff et al. (2022), and the majority of papers in the 2024 special issue of Zeitschrift für Psychologie, suggest CI is. For instance, in Imhoff et al. (2022) Figure 1 represents specific conspiracy belief as mean level endorsement of a collection of specific CTs (where the number of CTs ranges from from N=99, N=6, N=2). Similarly, Trella et al. (2024) note that “the construct we might call the conspiracy mentality is captured by the CMQ while the construct we might call belief in conspiracy theories is captured by aggregate scales such as the BCTI.” (my emphasis). The BCTI is consists of 15 specific CTs.

Thank you for bringing our attention to this potential point of confusion. Upon review, Imhoff et al., (2022) use the term “specific conspiracy beliefs” somewhat inconsistently. Although Panel A of Figure 1 in the Imhoff et al. (2022) article depicts the relation between a measure of what they call conspiracy mentality and the aggregate of agreement with 99 conspiracy theories—as mentioned in the above comment—Panels B and C specifically refer to conspiracy theories about the COVID-19 pandemic:

“The specific beliefs represent in Panel A the mean of the endorsement of 99 diverse conspiracy theories (Study 1, [9]), in Panel B the mean of six conspiracy theories about the COVID pandemic (all studies, [27]), and in Panel C the mean of two conspiracy theories about the COVID pandemic” (Imhoff et al., 2022; pg. 2)

Moreover, we cited Trella et al., (2024) because Study 1 of their paper showed that participants perceived *each item* of generic measures of conspiracist ideation like the Conspiracy Mentality Questionnaire as being more general than the specific conspiracy theories mentioned in the BCTI:

“… the findings confirm that participants see conspiracy mentality statements as more general than conspiracy theories themselves” (p. 11)

That said, to avoid any confusion with the inconsistent use of the term “specific conspiracy beliefs,” we have elected to use the term “event conspiracy beliefs” to make it clear that we are interested in conspiracy beliefs that help people make sense of individual events and have removed references to Imhoff et al., (2022) and Trella et al., (2024). Moreover, we have also reworked the introduction to avoid any controversy regarding different perspectives on the meaning of aggregated scales of real-world conspiracy theories versus measures that use more abstract items.

3. Measures of conspiracy ideation used in the two studies.
Study 2 measures CI using the CMQ, and Study 1 uses the GCBS. Although, it’s fair to say that the latter has been used as a conspiracy mentality measure by other authors, I think it is important to acknowledge that GCBS doesn’t measure exactly the same thing at the CMQ (and perhaps that might explain some of the differences in the findings across the two studies).

The items in the CMQ (and similar scales like the CMS) aren’t explicitly about conspiracies at all but about beliefs related to conspiracy belief, although not necessarily so. This probably explains why the distributions of scores on the CMQ and CMS differ from those for specific conspiracy theory belief (see Figure 1 of Imhoff et al., 2022).

If you look at the CMQ items they attempt to tap a variety of beliefs about authorities and political processes (such as powerful groups keeping secrets, things not being as they seem, coincidences hiding deeper truths and so on) whereas the GCBS measures beliefs about ‘high-level’ conspiracy theories that do not include detail about specific protagonists or events. The two types of measure are not abstract (an idea that is canvassed on p. 6 of the manuscript) in the same way. Sutton et al. (2024) claim that conspiracy mentality refers to an abstract, generalized political attitude. By contrast the GCBS is abstract in the sense that it measures beliefs about types or clusters of conspiracy theories.

We acknowledge that there has been increasing conversation regarding potential differences between measures of conspiracist ideation using generic statements. We have inserted text on pages 44 and 45 to bring awareness to these distinctions buttressed by a distinction made by a meta-analysis by Biddlestone and colleagues (2022) and reference the idea that the CMQ taps into a “generalized political attitude” according to Imhoff and Bruder (2014):

“Although not central to the current research, we briefly note that some have suggested that there are important differences between commonly employed measures of conspiracist ideation (Biddlestone et al., 2022). Measures like the Generic Conspiracist Beliefs Scale (Brotherton et al., 2013) that was employed in Study 1 tap into the degree to which people endorse generic assumptions about the typicality of conspiratorial activity in the world (e.g., “Some UFO sightings and rumors are planned or staged in order to distract the public from real alien contact”). In contrast, instruments like the Conspiracy Mentality Questionnaire (Bruder et al., 2013) that was employed in Study 2 tap into the broader ideological mindset of conspiracy mentality (sometimes referred to as a “generalized political attitude; Imhoff & Bruder, 2014) by assessing the degree to which people endorse aspects of conspiracist thinking (e.g., “I think that there are secret organizations that greatly influence political decisions”). For simplicity, and given the substantial empirical relation between these two measures (Swami et al., 2017), we refer to what is measured by each approach as conspiracist ideation—people’s general tendency to believe conspiracy theories. Moreover, the parallel findings that we observed with the two scales offers additional convergent validity for the conceptual framework.” (p. 45)

However, given their substantial overlap of these two measures (Swami et al., 2017) and the fact that our interest rests on the effects of event conspiracy beliefs, we do not discuss it further.

4. Measures of specific conspiracy theories used in the two studies.
The items used to measure specific conspiracy theories in Study 2 aren’t, strictly speaking, descriptions of specific conspiracy theories (although they are certainly ‘conspiracy-adjacent’). Neither of these items include explicit mention of a conspiracy or identify who the conspirators are or that the activity has been carried out for malicious reasons. I think it is worth pointing out this difference between the two studies. If you can find evidence to support the idea that people interpret statements like those used in Study 2 as conspiracy theory statements, that would be worth including.

Prior to the submission of this manuscript to the journal, we anticipated such concerns. Accordingly, our original submission presented data suggesting that people perceived the two items used to measure COVID-19 conspiracy beliefs as indeed tapping into conspiracy theories:

“To ensure that these items indeed concerned the endorsement of conspiracy theories rather than simply unwarranted claims, we recruited an independent sample of 497 participants to provide ratings of the extent to which the items were indicative of believing conspiracy theories. First, we defined the term conspiracy theory in the manner done in this manuscript, highlighting that “a conspiracy theory involves an assertion that dark forces are at work.” Next, participants were asked to consider whether believing various statements “would imply belief in a COVID conspiracy theory back during the pandemic.” They then rated the degree to which a person who endorsed each of the two items above would believe a conspiracy theory regarding COVID-19. We also asked participants to rate two items intended to serve as comparison points, one clearly expressing a conspiracy theory (“Lockdowns were put in place during the COVID-19 pandemic deliberately for the purpose of hurting the economy”) and one expressing a statement well-supported by evidence (“Social distancing is an effective method of preventing the spread of COVID-19”; Alfano & Ercolano, 2020). The four items above were rated using a seven-point scale ranging from *Not at all* (1) to *Very much so* (7), with *Moderately so* (4) serving as the midpoint. Participants believed that during the height of the pandemic, people were more than moderately likely to believe a COVID-19 conspiracy theory when they endorsed the ideas that COVID-19 was no more dangerous than the flu (*M* = 4.55, *SD* = 1.92), *t*(496) = 6.28, *p* < 0.001, *d* = 0.28, and that COVID-19 was developed in a lab in Wuhan (*M* = 5.34, *SD* = 1.88), *t*(496) = 15.92, *p* < 0.001, *d* = 0.71. As points of reference, participants also believed that (a) those who endorsed the item regarding lockdowns having been intended to hurt the economy were more than moderately likely to endorse a conspiracy theory regarding COVID-19 (*M* = 5.01, *SD* = 2.19), whereas (b) those who endorsed the evidence-supported item regarding the efficacy of social distancing were judged less likely to believe a COVID conspiracy theory (*M* = 2.94, *SD* = 2.08).” (pgs. 26-27)

List of Recommended Changes
1. Articulate what you think ‘CT belief controlling for CI and ET’ is (conceptually/theoretically-speaking) around pp. 9-10. DAGs might be useful.

As more fully articulated in point #1 above, we have expanded our discussion of the proposed causal relations underlying our research and have added a figure to depict these relations graphically.

2. Consider examining interactions or simple slopes to better understand how CT belief varies with CI (and ET).

We very much appreciate this suggestion, but given (1) the numerous analyses already presented in both the main text and supplement and (2) the focus of our research falling squarely on statistical control of the covariates rather than interactions between event conspiracy beliefs and the experience of economic turmoil and conspiracist ideation, we have elected to not conduct such analyses.

3. Related to (1), ensure that claims about mental health benefits is are clearly associated with ‘CT belief controlling for CM and ET’ and not CT belief simpliciter.

Once again, thank you for encouraging us to provide greater clarity regarding the interpretation of our multiple regression findings. As detailed above, we have inserted text on pages 22, 40, and 43 to communicate the appropriate interpretation of our findings.

4. Clarify what you mean by belief in specific conspiracy theories and conspiracy ideation. Ensure it is consistent with existing ideas (e.g., Imhoff et al., 2022) or that it is clear that you have a different take on this distinction from other authors.

We appreciate the encouragement to critically evaluate our use of terminology. As we explain in greater detail above, we seek to avoid confusion regarding alternate uses of the term “belief in specific conspiracy theories” by labeling our focal construct “event conspiracy beliefs” which are narrowly focused on conspiracy beliefs regarding an event.

5. Related to (4), clarify what you mean by conspiracy ideation/mentality.

We use the term “conspiracist ideation” to refer to the general tendency to engage in conspiratorial thinking, which is the most popular use of the term. Although there are ongoing discussions regarding the meaning of differences across measures of conspiracist ideation, our perspective is well-rooted in classic work in the literature.

6. Provide VIF/multicollinearity information and more detail about the standardisation procedure used on p. 15. I presume this means that z-scores were calculated rather than variables being mean-centred? Was this done to manage multicollinearity?

We have inserted text on pages 20 and 34 to communicate that multicollinearity is not an issue in our analyses, as indicated by VIF values well below the strict VIF = 5 threshold. We have also added text to indicate that we z-scored variables prior to entering them into the statistical models. Variables were standardized for the purpose of interpretation, not to deal with issues of multicollinearity. It is our understanding that standardizing variables is sometimes used to manage issues of multicollinearity when dealing with interaction terms, but as can be verified using our data, standardization does not impact VIF values in multiple regression analyses.

7. Provide zero-order correlation information.

We have added correlation matrices on pages 18 and 33.

8. Clarify paragraph 1 on p. 10. I don’t understand “may fare better than those left with an explanation whose scope seems to not match the large scale of the issue”. What is this alternative (non-conspiracy?) explanation alluded to here? Why is ‘scope matching’ an issue? Why doesn’t conspiracy ideation provide adequate ‘scope’? And how does the claim in the final sentence relate to the rest of the paragraph? I found this paragraph particularly difficult to make sense of and, given it is part of the justification for ‘controlling for’ CI, seems to be a key part of the rationale for the overall analytic approach taken in the paper.

Thank you for raising concerns regarding this paragraph. We agree that we could be clearer in our communication. We have edited this paragraph in several ways. First, we have clarified the non-conspiratorial explanations to which we refer. Second, we have contrasted the conspiratorial explanations with non-conspiratorial explanations in several ways and suggest how conspiracy-based explanations for events may be less stressful. Third, we explain how proportionality bias may present an issue for non-conspiratorial explanations. As seen on page 13:

“The need to account for the experience of turmoil when examining potential benefits of event conspiracy beliefs seems relatively straightforward. However, readers may wonder why we propose that an event conspiracy belief can have benefits for people’s level of stress and well-being, while the general tendency to believe conspiracy theories does not. We contend that, during a period of uncertainty and confusion like that created by the COVID-19 pandemic, individuals who endorse event-related conspiracy theories—logically flawed as they may sometimes be (Albarracin et al., 2021)—may fare better than those left without a clear causal explanation (Giacomantonio et al., 2022). That is, non-conspiracy explanations, such as random chance or human error, mean the world is unpredictable, which is itself stressful (Grupe & Nitschke, 2013). When a group of people is conspiring, the world may seem less unpredictable. Moreover, such non-conspiracy explanations may feel insufficient or trivial in the face of a global crisis due to proportionality bias—the tendency to believe that large effects stem from large causes (Bortolotti, 2023). Thus, a global shutdown may be more easily understood as being caused by the evildoings of global elite than by a virus spread in a wet market. Conversely, higher levels of conspiracist ideation are unlikely to furnish such an overall benefit to the believer. The frequent tendency to see dark forces operating in numerous domains of one's life and the perseveration on those forces that accompany higher levels of conspiracist ideation is obviously stressful, as noted earlier (e.g., Freeman & Bentall, 2017).”

9. p. 16 – I’d be inclined to avoid talking about marginal significance – I don’t think there it makes a great deal of difference it you just say that there’s no significant relationship between conspiracy ideation and stress for Study 1.

We understand the hesitation to use the term “marginal significance” and agree that this issue does not make a great deal of difference to the aim of the paper. However, given the accumulated literature on conspiracist ideation and well-being, we think it is important to take a more cumulative approach to evidence evaluation in which even non-significant results can increase our confidence in a general pattern of findings. That is, although conspiracist ideation does not significantly predict stress in Study 1, the direction is very much consistent with prior work.

10. Similarly p = .09 is called marginally significant on p. 28. I’d be inclined to simply say that CT + CM does not predict negative symptomology. Perhaps use the same approach to describe this kind of finding as has been done for the p =.15 finding at the bottom on p. 28.

We appreciate bringing attention to how we label effects as “marginally significant.” We use a cutoff of *p* < 0.10 to describe findings as marginally significant. Consistent with this rule, we have elected to retain our labeling of the effects above.

11. Note that GCBS and CMQ don’t measure exactly the same thing although they seem to correlate reasonably well (e.g., in Swami et al., 2017) – as does pretty much everything in the conspiracy world it seems.

Thank you for this suggestion. As discuss previously, we have added text on page 45 to draw the reader’s attention to perspectives on the meaning of the indices provided by those two measures. We do not delve deeper into this issue given that it does not bear directly on the purpose of our research.

12. Note that the ‘conspiracy-adjacent items’ in Study 2 aren’t explicit specific conspiracy theories (like those used in Study 1). They don’t describe a conspiracy or conspirators that is done for malicious reasons.

We anticipated such concerns regarding the measures of COVID-19 conspiracy beliefs in Study 2. As such, we present analyses suggesting people do perceived the two items used to measure COVID-19 conspiracy beliefs tap into conspiracy theories on pages 26 and 27.

13. The timing of the measures in Study 2 raises some questions. I’m not sure if it sufficiently clear that the Wave 3 analyses use ‘past measures’ of economic turmoil, ideation, and CT belief that are 3-4 months older than the Wave 3 subjective wellbeing measure. That is, neither the Wave 3 or the Wave 5 analyses examine current levels of economic turmoil, ideation, and CT belief. We simply don’t know if the Wave 1 and Wave 2 measures are a reasonable measure of current economic turmoil, ideation, and CT belief for the analyses.

We view the lagged nature of the analyses in Study 2 as a strength of our research. Indeed, the fact that our key predictors are measured at earlier timepoints allows us to prospectively predict later well-being. We believe that the original submission made such differences in time of measurement clear by labeling all measures by the wave in which they were measured in the Methods section of Study 2 (e.g., “Belief in COVID-19 Conspiracy Theories – Wave 2” on page 26). Moreover, we mention the prospective nature of the research on pages 39, 40, and 42 as we analyze our results. Although prospective analyses like those in Study 2 are not perfect, they do provide some window into temporal precedence, which is a key criterion for establishing causality.

**References**

Allen, M. S., Iliescu, D., & Greiff, S. (2022). Single item measures in psychological science. *European Journal of Psychological Assessment, 38*(1).

Barkun, M. (2013). *A culture of conspiracy: Apocalyptic visions in contemporary America* (Vol. 15). University of California Press.

Bayliss, D., Olsen, W., & Walthery, P. (2017). Well-being during recession in the UK. *Applied Research in Quality of Life*, *12*, 369-387.

Brotherton, R., French, C. C., & Pickering, A. D. (2013). Measuring belief in conspiracy theories: The generic conspiracist beliefs scale. *Frontiers in Psychology*, *4*, 279.

Darlington, R. B., & Hayes, A. F. (2016). *Regression analysis and linear models: Concepts, applications, and implementation*. Guilford Publications.

Diener, E. (1984). Subjective well-being. *Psychological Bulletin*, *95*(3), 542–575.

Diener, E., Oishi, S., & Tay, L. (2018). Advances in subjective well-being research. *Nature Human Behaviour*, *2*(4), 253-260.

Douglas, K. M., Uscinski, J. E., Sutton, R. M., Cichocka, A., Nefes, T., Ang, C. S., & Deravi, F. (2019). Understanding conspiracy theories. *Political Psychology*, *40*, 3-35.

Imhoff, R., & Bruder, M. (2014). Speaking (un–)truth to power: Conspiracy mentality as a generalised political attitude. *European Journal of Personality*, *28*(1), 25–43.

Imhoff, R., Bertlich, T., & Frenken, M. (2022). Tearing apart the “evil” twins: A general conspiracy mentality is not the same as specific conspiracy beliefs. *Current Opinion in Psychology*, *46*, 101349.

Park, N., Heo, W., Ruiz-Menjivar, J., & Grable, J. E. (2017). Financial hardship, social support, and perceived stress. *Journal of Financial Counseling & Planning*, *28*(2).

Stallings, M. C., Dunham, C. C., Gatz, M., Baker, L. A., & Bengtson, V. L. (1997). Relationships among life events and psychological well-being: More evidence for a two-factor theory of well-being. *Journal of Applied Gerontology*, *16*(1), 104-119.

Swami, V., Barron, D., Weis, L., Voracek, M., Stieger, S., & Furnham, A. (2017). An examination of the factorial and convergent validity of four measures of conspiracist ideation, with recommendations for researchers. *PLOS ONE*, *12*(2).

Swami, V., Chamorro‐Premuzic, T., & Furnham, A. (2010). Unanswered questions: A preliminary investigation of personality and individual difference predictors of 9/11 conspiracist beliefs. *Applied Cognitive Psychology*, *24*(6), 749-761.

Tausig, M., & Fenwick, R. (1999). Recession and well-being. *Journal of Health and Social Behavior, 40*(1), 1-16.

Trella, C., Sutton, R. M., & Douglas, K. M. (2024). Semantic and Causal Relations Between the Conspiracy Mentality and Belief in Conspiracy Theories. *Zeitschrift Für Psychologie*, *232*(1), 7–17.
