# Supplementary material for: Is there anything good about conspiracy beliefs? Belief in COVID-19 conspiracy theories is associated with benefits to well-being
Source: PLoS One. 2025 Mar 21;20(3):e0319896. doi: 10.1371/journal.pone.0319896 (PMC11927887; doi:10.1371/journal.pone.0319896)
Supplement: Attachment — Submitted filename: Response to Reviewers_R2.docx [file pone.0319896.s004.docx]

**Response to reviewers**

We would like to thank the editor and reviewers for their favorable reactions to our manuscript and for taking the time to provide helpful comments. We have carefully considered each comment, and have made multiple revisions to the manuscript. As was the case with the earlier submission, our manuscript has greatly benefited from the feedback. We reproduce each comment offered in the action letter below in blue font, and provide responses in this black font. All page numbers referenced correspond to the “Manuscript with Track Changes” document.

**Editor:**

Overall, all three reviewers acknowledge the improvements made to the manuscript and appreciate the revisions. However, since two reviewers recommend minor revisions, particularly concerning the conceptualization of CTs, I kindly ask that you further refine the relevant sections and address their comments.

We were glad to hear that our revision was well-received. We provide in-depth responses to the reviewer comments in the pages below.

**Reviewer #2:**

My major criticism of the original manuscript was in relation to the issue of causal inferences. In response to this, you have provided causal justifications for the control variables, been explicit about the intent to draw causal inferences, and considered additional sources of evidence for causal effects you could provide. On the latter point you were able to provide a propensity scores analysis. These are all great improvements. What I'd like to see now is a re-calibration of specific conclusions made in the title, abstract, and main body to ensure that they are calibrated to the evidence for causal effects (which is only tentative), and to be consistent with the (accurate) caveat that "strong causal claims are unwarranted". I apologise for not making this expectation explicit in my initial review. Some of the specific claims I noticed that still make causal inferences without highlighting uncertainties include:

Thank you for bringing attention to this issue. Points 1-6 below reference different quotes that can be recalibrated to better communicate the non-experimental nature of the data. For each point, we provide the edited text in its broader context and its associated page number.

1. "Belief in COVID-19 conspiracy theories can provide benefits to well-being". (It's a really nice title, but in this form it just isn't warranted by the evidence in the paper)

“Is there anything good about conspiracy beliefs? Belief in COVID-19 conspiracy theories is associated with benefits to well-being” (p. 1)

2. "The current research provides evidence for benefits of an event conspiracy belief" (abstract)

“The current research provides correlational evidence for a link between well-being and an event conspiracy belief by teasing apart its effect from (1) the influence of experiencing turmoil that nudges people toward believing the event conspiracy theory in the first place and (2) conspiracist ideation—the general tendency to engage in conspiratorial thinking.” (p. 2)

3. "conspiracy beliefs do provide at least temporary intrapersonal benefits" (abstract)

“These findings suggest that despite their numerous negative consequences, event conspiracy beliefs are associated with at least temporary intrapersonal benefits.” (p. 2)

4. "To our knowledge, this is the first instance in which a beneficial intrapersonal effect of believing conspiracy theories on stress has been documented." (p. 22)

“To our knowledge, this is the first instance in which a positive relation between believing conspiracy theories and stress has been documented.” (p. 22)

5. "Most importantly, this study [2] provides evidence that believing in specific conspiracy theories can have benefits for the believer—conceptually replicating the results of Study 1" (p. 39).

“Most importantly, this study provides evidence that believing in specific conspiracy theories *can* be associated with boosts in well-being—conceptually replicating the results of Study 1—and clarifies that such relations are specific to increased contentment for the believer.” (p. 40)

6. "Putting these findings together suggests that the benefits of believing specific conspiracy theories operate by boosting positive affect" (p. 40-41).

“Putting these findings together suggests that any association between believing specific conspiracy theories and well-being is restricted to positive affect—which may make people feel more capable of dealing with the threats they face—but only for a limited amount of time.” (p. 42)

There could be others I've missed. I know that there's some great nuanced thinking about causality in the response letter, and in some other parts of the manuscript, and I'm definitely not suggesting you just delete all these sentences. But considered in isolation these are unambiguous "strong causal claims" that don't acknowledge uncertainty. Inevitably, it will be these strong causal claims that will be picked up and amplified by journalists and researchers citing your work. So please present *every* causal inference in such a way that it acknowledges relevant uncertainties and/or assumptions you're making.

We reviewed the manuscript thoroughly to find other instances in which we may be able to recalibrate the claims. We list such instances below:

Original quote: “However, the contentment benefit conferred by an event conspiracy belief recedes over time.”

Revised quote: “However, the relation between COVID-19 conspiracy belief and contentment diminishes in size over time.” (p. 2)

Original quote: “In summary, we propose that believing conspiracy theories may indeed have benefits for the believer, but that uncovering these benefits requires disentangling the influence of endorsing specific conspiracy theories from…”

Revised quote: “In summary, we propose that believing conspiracy theories may indeed be associated with benefits for the believer, but that uncovering these associations requires disentangling the influence of endorsing specific conspiracy theories from…” (p. 14)

Original quote: “In Study 2, we extended our prior findings by examining how belief in COVID-19 conspiracy theories influences different aspects of well-being and how these relations change over time.”

Revised quote: “In Study 2, we extended our prior findings by examining how belief in COVID-19 conspiracy theories relates to different aspects of well-being and how these relations change over time.” (p. 38)

7. In addition, I'd like to see that paragraph about causal limitations in the discussion section expanded into something a bit more comprehensive. What assumptions do readers need to make to interpret your beta values as unbiased estimates of causal effects? How realistic are those assumptions? What would happen if they're breached? E.g., one such assumption is that aside from conspiracist ideation and turmoil there exists no other third variable that affects both belief in specific conspiracy theories and wellbeing...

Thank you for encouraging us to expand on the conditions necessary to interpret our results as unbiased estimates of causal effects. We have taken this opportunity to go beyond the suggestion and discuss the criteria for establishing causality more generally. We follow this exposition by describing how our data fit with these criteria:

Establishing causality requires demonstrating evidence of covariation, temporal precedence, as well as ruling out plausible alternative explanations (Shadish et al., 2002). Across two studies, we provide evidence of covariation between event conspiracy beliefs and well-being. Although not definitive, the prospective nature of Study 2 provides some evidence regarding temporal precedence of the proposed relation between event conspiracy beliefs and well-being. Lastly, interpreting our results as unbiased estimates of the causal effect of event conspiracy beliefs on well-being requires ruling out potential third variables. Naturally, a failure to rule out potential third variables would bias the estimates of the relation between event conspiracy beliefs and well-being, increasing the possibility of a spurious correlation. Although this research cannot definitively rule out all alternative explanations, it does leverage multiple regression and propensity score weighting analyses to rule out the most relevant situational, dispositional, and demographic variables as competing explanations based on the current state of the literature. In so doing, we furnish evidence that is consistent with the idea that believing specific conspiracy theories can have benefits for well-being. While it is possible that confounders other than those tested exist, we are unaware of likely candidates for the role. Nevertheless, it should be kept in mind that the current findings represent merely a step, albeit an important one in our view, in building a case for a causal relation. The research presented here establishes a positive association between event conspiracy beliefs and well-being. (pgs. 44)

8. In my first review I pointed out that the pattern of findings across studies was consistent with your interpretation, but also consistent with an interpretation that the evidence of effects is tentative and inconsistent. I really appreciate your response but I'm afraid I remain unconvinced! In Study 2 there are 4 wellbeing outcomes (negative symptomology and contentment, both at waves 3 and 5). Only one of these 4 is significantly & positively predicted by belief in conspiracy theories (contentment at wave 3). And this finding isn't quite consistent with Study 1, because Study 1 used stress as an outcome measure, which is negative symptomology, not contentment. The positive coefficient in Study 2 is occurring only in the (unusual) circumstance of a bivariate positive correlation between belief in CTs and contentment, which isn't very consistent with the "suppressor variables" idea articulated earlier in the paper. As it stands, this paper presents only tentative evidence for positive effects of belief in CTs (both due to the causal inference issue and the not-entirely-consistent results). That *doesn't* mean it's not a useful contribution to the literature, and I'm 100% ok with the fact that you have decided an additional study isn't feasible... but I'd like to see that discussion section show a bit more acknowledgment of how much uncertainty remains until more followup work is done.

We appreciate the encouragement to better communicate the nature of our results. First, we would like to clarify that stress is not necessarily considered negative symptomology. Although we can certainly understand the link being made in the comment given the uncomfortable state that characterizes feeling stressed, the stress construct is recognized as being related to but distinct from well-being by the leading figures in that area of research. As indicated in the original revision, stress is used as a proxy for well-being in Study 1. To ensure that there is no confusion about the nature of the stress measure, we have added a sentence clarifying the nature of the construct and its relation to well-being:

As a proxy for well-being, we assessed people’s reports regarding the general level of stress they were currently experiencing. Stress refers to an individual’s appraisal of the demands placed on them in relation to their resources (Moskowitz, 2007), and is related to but distinct from well-being (Ng & Diener, 2022). (pg. 15)

That said, we do see value in better communicating the alternative interpretation of our results articulated above and the need for independent validation. We have inserted text into page 42 to communicate these points:

We interpret the results as providing robust evidence for our hypothesis of a relation between event conspiracy beliefs and well-being. However, the nature of the results in Study 2—in which COVID-19 conspiracy beliefs selectively predict contentment in the short-term but not the long-term—may also be interpreted as indicating that the relation between event conspiracy beliefs and well-being is at least somewhat tentative. As with all research documenting novel relations, independent replication is required.

9. Re. my original point 6, I think you make good points about the merits of single items. However, the fact remains that your control variables all inevitably have at least *some* measurement error attached, and your regression models assume they don't. Even without any other confounds this would be enough to cause the estimates of the focal causal effects to be biased (see Westfall & Yarkoni, 2016). I do think you need to acknowledge this in the manuscript.

We thank the reviewer for bringing attention to this issue. Before directly addressing the key point of this comment, we would like to point out that we use Structural Equation Modeling to ensure that the multiple regression analyses reported in the manuscript are robust to tools that take into account measurement error for multi-item measures, which we report in the Supplemental Material. Of course, single-item measures are treated as observed variables in SEM, and thus, the concern regarding the measurement error associated with them is not addressed by these models. We would like to note, however, that when using a large sample like that of Study 2, the introduction of measurement error almost exclusively reduces the effect size of the relation in question: “In the large-N scenario, adding measurement error will almost always reduce the observed correlation between x and y (see the figure, left panel).” (p. 585; Loken & Gelman, 2017). That said, we do see value in directing future research to circumvent such issues by using multi-item measures. We have inserted text on page 42 to encourage future replication attempts to use multi-item measures to assess economic turmoil:

Despite finding robust associations between economic turmoil and well-being, one valuable aspect of a future replication attempt would be the measurement of economic turmoil using a multi-item measure to ensure that this construct is assessed more comprehensively and measurement error can be modeled.

Presentational points:
10. Figure 1 is a very useful addition. The arrows ppointing to wellbeing don't render clearly in the pdf, so it'd be worth double-checking they look ok during copyediting.

Thank you for bringing our attention to this potential issue. We will attach the image file to our submission to ensure that the image is rendered in high quality.

11. P. 29, the 2 in the chi-square symbols should be superscript.

Thank you for finding these errors. The 2 in the chi-square symbol has been changed into a superscript.

**Reviewer #3:**

The authors have done a nice job attending to many of the comments from the reviewers. There are two areas that I think could still do with some attention:

1. Conspiracy item used in Study 2.

“COVID-19 is no more dangerous than the common cold" isn’t a conspiracy theory item - at best we can call it an empirically unsupported claim that underpins a number of conspiracy theories. I think it would be appropriate for the authors to describe it thus. The survey (described on p. 26 of the manuscript) asking people whether they think someone who endorses the claim believes a conspiracy doesn’t alter this fact. I suspect quite a few non-conspiracy claims might result in a similar type of response (e.g., “January 6th protestors had every right to enter the Capitol” or even “Trump tells the truth” or “There are some things that people see in the sky that aren’t easily explainable”). Endorsing such claims might be something you’d expect of someone who believes particular conspiracies but that doesn’t make the claims themselves conspiracy theories and, more importantly, it isn’t strong evidence that a participant who endorses them actually believes a particular conspiracy. Moreover, the claim doesn’t even ‘pick out’ a single conspiracy theory - it is consistent with a variety of conspiracies as well a number of non-conspiracy beliefs - someone could believe this claim without thinking that it involves secrecy or coverups, or that a group has malevolent intentions.

We appreciate the opportunity to be more precise about the nature of the item referenced above. To accommodate the suggestion above, we have taken the step of referring to both items concerning COVID-19 as tapping into empirically unsubstantiated claims that underpin COVID-19 conspiracy beliefs. These changes are reflected in (1) a new heading for the section describing these measures that reads “Belief in Empirically Unsubstantiated Claims/COVID-19 Conspiracy Theories – Wave 2” and (2) new text inserted on page 26:

Our reliance on publicly available data necessitated the use of measures of empirically unsubstantiated claims as proxies for COVID-19 conspiracy beliefs. Specifically, we used two self-report items focused on people’s endorsement of the ideas that (1) the severity of the COVID-19 pandemic was exaggerated (“Coronavirus is actually no more dangerous than the common flu.”) and (2) COVID-19 was deliberately created (“Covid-19 was developed in a lab in Wuhan, China.”). Both items were rated using a slider scale ranging from *Do not believe at all 0%* to *Completely believe 100%.* These two items were selected for use in Study 2 due to their similarity to those used to measure belief in COVID-19 conspiracy theories in Study 1. Although the two items composing this scale are more modestly correlated than in Study 1 (*r* = 0.25), the results remain substantively unchanged when using each individual item as a predictor instead of the composite measure (see Supplemental Material for full details).

We acknowledge that the items above—particularly those involving the severity of COVID-19—more clearly assess endorsement of empirically unsubstantiated claims that underpin COVID conspiracy beliefs than conspiracy theories per se. That said, we wished to test whether these unsubstantiated claims could be interpreted as being indicative of conspiracy theories.

2. Clarify the distinction between conspiracy ideation (CI) and (event) conspiracy theory belief (CT) that is central to the claims of the paper.

My comments in the initial review were intended to be about the conceptual distinction being made (or implied) in the research, rather than a point about the interpretation of analyses - however, I can see why they were interpreted that way. Apologies for my lack clarity. I should also have made it clearer that my main concern is with the idea that ‘CT controlling for CI’ makes good conceptual sense. (the economic turmoil variable seems more theoretically tractable to me). From a statistical point of view the paper seems fine to me (although I agree with Reviewer 2’s points about the need to be cautious about drawing causal conclusions from observational/correlational analyses). The authors provide evidence for CI and CT being statistically different constructs (using factor analysis) despite them being quite strongly correlated (something that has been observed before of course - Imhoff et al., 2022 explore some possible reasons why this is the case). The majority of the regression analyses show that the CI measure acts as a suppressor on CT measure-wellbeing relationships.

To address the central issue raised above, we have attempted to clarify our position regarding the conceptual interpretation of our key predictor in the context of multiple regression models by adding text on pages 44 and 45:

People higher in conspiracist ideation would be expected to endorse COVID-19 conspiracy theories to a greater extent by the nature of their general predisposition toward such explanations. Despite this predisposition, aspects related to the specific situation and how it impacts an individual contribute to the degree to which they endorse an event conspiracy theory. Indeed, individuals may experience situational pressures that spur the development of an event conspiracy belief (Biddlestone et al., in press; Jetten et al., 2024; Pica et al., 2024), even those relatively low in conspiracist ideation (Granados Samayoa et al., 2021)—which is how we view the postulated role of economic turmoil in the present work. Conceptually, we interpret belief in COVID-19 conspiracy theories when taking conspiracist ideation into account as representing people’s belief that the events surrounding COVID-19 involved a conspiracy theory that are divorced from their general tendency to believe conspiracy theories and their associated constituents like anti-elitism and distrust of authorities. That is, we seek to isolate the aspect of endorsement of COVID-19 conspiracy theories that is unique to people’s reactions to the COVID-19 pandemic rather than their general tendency to accept conspiratorial explanations. In effect, conspiracist ideation offers what might be considered a default level of acceptance of any given event conspiracy belief, but variability around that default is to be expected. For example, an individual whose conspiracist ideation focuses largely on distrust of government officials may accept a conspiratorial belief regarding a specific event involving the government readily, but may be less prone to accept a conspiratorial belief regarding a scientifically-supported medical practice.

We would like to note that controlling for a dispositional construct when interested in isolating the specific influence of a situational construct—the very aim of this research—is an accepted practice. For example, Story and colleagues (2007) were interested in examining age differences in positive sentiment override—the more positive assessment of spouse's affiliative behavior than is warranted by observed behavior—when taking into account dispositional optimism. Certainly, optimism predicts positive sentiment override robustly. However, there remains meaningful variance in positive sentiment override despite being divorced from the general tendency to see things in a positive light. Similarly, in research published in *PLOSOne*, Salo and colleagues (2020) tested whether depression and empathy towards one’s children when holding general empathy constant. Once again, general empathy and empathy toward one’s children are strongly correlated, yet it is accepted that controlling for general empathy while examining associations with the more specific instance of empathy is a valid practice in which the more specific construct is not stripped of its meaning.

So, to repeat, my worry is that there’s still a lack of conceptual clarity about how we should make sense of these findings. I think that the discussion on pages 10-13 of the new manuscript goes a long way towards providing the kind of substantial theoretical rationale that is needed. The authors seem to see the CI-CT distinction differently from authors like Imhoff do, where CI is understood as a mechanism (disposition, propensity, “distal predictor”) that “largely determines” (in the words of Imhoff & Bruder, 2014) belief in specific CTs. Framed this way it doesn’t make much sense (to me) to include both CI (mechanism/part cause) and CT (effect) in a model of predictors of wellbeing. It is difficult to know how we can interpret CI as a suppressor under this reading.

We are delighted to hear that the reviewer found our discussion to be valuable. As noted in the point above, we have bolstered our interpretation of the conceptual interpretation of belief in COVID-19 conspiracy theories on pages 44 and 45. That said, we would like to address the comment regarding Imhoff and Bruder’s (2014) suggestion that conspiracist ideation “largely determines” belief in specific conspiracy theories. To start, we note that Imhoff and Bruder (2014) do not present any direct evidence regarding the relative contribution of situational and dispositional factors to the prediction of specific conspiracy beliefs, instead focusing on distinguishing it from other constructs (e.g., right-wing authoritarianism) and exploring its consequences (e.g., prejudice against high-power groups). More to the point, far from viewing conspiracist ideation as a rigid, unidirectional determinant of specific conspiracy beliefs, a recent paper by Imhoff (2024) comments on the idea that there might be a general propensity to believe conspiracy theories in the following manner (something akin to a *g* factor for endorsement of conspiracy theories):

Nera now criticizes this view as intuitively suggesting a unidirectional causal relation from conspiracy mentality as a general propensity to the endorsement of specific conspiracy theories. I beg to differ here – claiming a relatively stable propensity does not imply a unidirectional causal claim.

As documented by the gateway conspiracy effect (Granados Samayoa et al., 2022), belief in event conspiracy theories can also lead to increases in conspiracist ideation. Thus, it is well accepted that conspiracist ideation and belief in more narrow, event conspiracy beliefs can exert reciprocal effects on each other. From Imhoff (2024):

The inductive perspective would imply that some people find very specific conspiracy theories highly plausible (for whatever reason) and use these experiences of plausible conspiracy theories to induce a general rule that many (or most) events or phenomena can be explained by plots hatched in secret. Such a perspective allows for the interesting research question of the characteristics and situational context of such gateway conspiracy theories that initiate this process. From a deductive perspective, people have a certain belief about how the world operates in principle (e.g., most things are determined by secret plans of a few powerful agents)and use this belief as a schematic foil against which world events are interpreted… Importantly, however, these two perspectives are not mutually exclusive, and both models of causation can be true: conspiracy mentality as an over-generalization of specific beliefs and as an interpretative foil to make sense of (new) aspects of the world.

This in no way implies that they should not be entered into a statistical model. Indeed, it is this interrelation that necessitates that we separate them statistically.

By contrast with this ‘mechanism-effect view’, the authors argue that CI is a construct associated with chronic, maladaptive rumination whereas specific/event CT belief is an adaptive attempt at explanation that makes sense of troubling phenomenon in a satisfying fashion. These differences explain why wellbeing is related to the two constructs in different ways. This is an interesting idea but I do wonder whether it involves a novel redefinition of CI (and CT for that matter - now we are treating event conspiracy beliefs as something different from how other researchers talk about specific conspiracy beliefs) in a slightly question begging way - that is, the constructs get redefined in order to justify the analyses.

We thank the reviewer for encouraging us to strengthen our discussion of why conspiracist ideation may prove harmful to well-being. To this end, we have added text citing research suggesting that conspiracist ideation is associated with maladaptive coping strategies, which has been linked to poorer well-being:

Beyond a general focus on dark plots underlying a variety of events, conspiracist ideation may be unlikely to confer psychological benefits due to its association with learned helplessness and maladaptive coping strategies that are known correlates of poorer well-being (e.g., Farhart et al., 2021; Marchlewska et al., 2022). (p. 13)

The discussion on p. 13 of the edited manuscript speculating about why a conspiracy explanation (event conspiracy belief) might be less stressful than a non-conspiratorial alternative (that posits an unpredictable world and/or a cause that is not in proportion with the effect) is intriguing (although it does seem at odds with the findings of Stojanov & Halberstadt, 2020 who found little evidence that perceived lack of control predicted conspiracy belief).

We are glad that our added text was intriguing. We would like to clarify the statement above suggesting that Stojanov and Halberstadt (2020) “found little evidence that perceived lack of control predicted conspiracy belief.” The discussion about why a conspiracy explanation may be less stressful than a non-conspiratorial alternative referenced above is consistent with the work of Stojanov and Halberstadt (2020), as these authors found no relation between perceived lack of control and conspiracist ideation, but did find a relation between perceived lack of control and belief in specific conspiracy theories:

“For the moderators with two levels, we ran separate analyses on effect size using each potential moderator as an independent variable in turn. These analyses revealed only a significant effect of DV operationalization, β = −0.14, 95% CI [−0.26, −0.02], p = .02, such that the threats to control increased beliefs in specific conspiracy theories ( d = −0.11, 95% CI [−0.20, −0.02], p = .02), but not generic conspiracy theory beliefs (d = 0.03, 95% CI [−0.07, 0.14], p = .48).”

The following paragraph (lines 17-20) that speculates about how CI might result in lower wellbeing/higher stress also sounds plausible - yet together, the two do seem at odds with each other. That is, CI predicts poorer wellbeing because it’s stressful to ruminate about dark forces affecting ones life - but, for some reason, ruminating about specific dark forces affecting a specific event in ones life has the opposite effect.

Could we not (a priori) flip the arguments around and argue that CI should be LESS stressful than the alternative (having a propensity to think the world is unpredictable and often big things happen because of small causes)? And could we not also argue that event CT belief should be MORE stressful (than other kinds of explanations) because it is stressful to believe that dark forces are affecting ones life? It seems to me that, the proposed explanation of the different relationships CI and CT have with wellbeing, needs to be bolstered by, for instance, ruling out the possibility that CI could be MORE satisfying and LESS stressful than alternative explanatory propensities.

We thank the reviewer for this comment. Although one could flip the predictions in the manner suggested above, the current state of the literature does not provide strong rationale for doing so. As we note on page 8, conspiracist ideation has been consistently associated with undesirable psychological outcomes:

Conspiracist ideation—the general tendency to engage in conspiratorial thinking—appears to be similarly associated with an increase in learned helplessness, greater stress, and poorer well-being, among a variety of other undesirable mental health outcomes (e.g., Farhart et al., 2021; Freeman & Bentall, 2017).

We were unable to formulate a theoretical argument as to why a beneficial effect of conspiracist ideation could be suppressed, and thus, we did not advance this hypothesis. On the other hand, as we explain on pages 8 and 9 of the main text, there is reason to suspect that believing event conspiracy theories is associated with well-being when taking into account the forces that operate simultaneously with the development of event conspiracy beliefs—experiencing economic turmoil and conspiracist ideation—the pattern for which we find support.

**References**

Granados Samayoa, J. A., Moore, C. A., Ruisch, B. C., Boggs, S. T., Ladanyi, J. T., & Fazio, R. H. (2022). A gateway conspiracy? Belief in COVID-19 conspiracy theories prospectively predicts greater conspiracist ideation. *PLoS One*, *17*(10), e0275502.

Imhoff, R. (2024). On the usefulness of the conspiracy mentality concept. *Zeitschrift für Psychologie*, 232(1), 59–63.

Loken, E., & Gelman, A. (2017). Measurement error and the replication crisis. *Science*, *355*(6325), 584-585.

Story, T. N., Berg, C. A., Smith, T. W., Beveridge, R., Henry, N. J. M., & Pearce, G. (2007). Age, marital satisfaction, and optimism as predictors of positive sentiment override in middle-aged and older married couples. *Psychology and Aging, 22*(4), 719–727.
